# Modelling forest carbon stocks on the Canary Islands

**DOI:** 10.1186/s13021-026-00488-4

**Published:** 2026-07-27

**Authors:** Rüdiger Otto, Juan José García-Alvarado, Elena Rocafull, Natalia Sierra Cornejo, Severin D. H. Irl, Felipe Rodríguez Arvelo, Ricardo Ruíz-Peinado, José María Fernández-Palacios, Lea de Nascimento

**Affiliations:** 1 Departamento de Botánica, Ecología y Fisiología Vegetal, Universidad de La Laguna (ULL), Avda. Astrofísico Francisco Sánchez, s/n, La Laguna, Canary Islands, San Cristóbal de La Laguna, 38200 Spain; 2 Institute of Physical Geography, Department of Geoscience, Johann Wolfgang Goethe University, Altenhoeferallee 1, D-60438 Frankfurt am Main, Germany; 3 Department of Forest Dynamics and Management, Institute of Forest Science, ICIFOR-INIA), CSIC, Ctra. A Coruña, km 7.5, Madrid, 28040 Spain; 4 Island Ecology and Biogeography Group, Instituto Universitario de Enfermedades Tropicales y Salud Pública de Canarias (IUETSPC), Universidad de La Laguna (ULL), Avda. Astrofísico Francisco Sánchez, s/n, La Laguna, Canary Islands, San Cristóbal de La Laguna, 38200 Spain

**Keywords:** Canary Islands, Forest carbon stocks, LiDAR, Machine learning, Laurel forest, Sentinel-2, National Forest Inventory, Carbon mapping

## Abstract

**Background:**

Forest carbon mapping is crucial for sustainable forest management, climate mitigation, biodiversity conservation, ecosystem service provision and land-use planning. Carbon stocks have been studied at regional to global scales and across various biomes. However, island-wide studies of carbon stocks and carbon mapping remain limited. Here, we present the first high-resolution (50 m) spatial mapping of forest carbon for the Canary Archipelago. Combining structural field data from the Spanish National Forest Inventory plots with airborne laser scanning, Sentinel-2 multispectral satellite data and fine-scale interpolated climatic variables, we modeled total, aboveground and belowground carbon density of 18 forest types, using machine learning approaches, including Boosted Regression Tree and Random Forest models.

**Results:**

Forests across the Canary Islands store an estimated 10.26 Tg of carbon. Canarian pine forests contain the largest carbon pool (57%) due to their extensive distribution area, whereas mature laurel forests exhibit exceptional carbon densities. In humid laurel forests, average carbon densities reached 413.2 ± 149.5 Mg C ha⁻¹, exceeding previous regional estimates and approaching levels of primary tropical forests. The high spatial heterogeneity of carbon densities across forest types and islands was best explained by structural stand attributes, such as tree canopy cover and volume, and climatic factors with carbon stocks being more strongly associated with moisture than with temperature. Both statistical modelling approaches performed similarly in terms of efficiency, accuracy and error statistics, and the selection of the best model depended on the specific island.

**Conclusions:**

Our approach integrates field data with advanced remote sensing tools and machine learning algorithms to produce a high-resolution and accurate carbon map of topographically complex oceanic islands. We show that the Canary Islands contain exceptionally high total carbon densities, particularly within the mature, humid laurel forests of La Gomera, and identify structural attributes and water availability as the main drivers of spatial variation in carbon stocks. This assessment provides a baseline for biodiversity conservation, nature-based forest management, ecological restoration and regional climate policy towards carbon neutrality in island ecosystems.

**Supplementary Information:**

The online version contains supplementary material available at 10.1186/s13021-026-00488-4.

## Background

Forests represent important components of the global terrestrial carbon cycle, acting as major sinks that sequester atmospheric carbon dioxide through photosynthesis and store it within plant biomass and soil organic matter [1–3]. The sustainable management of forest resources has become increasingly relevant for mitigating the impact of climate change. Forest carbon mapping is a useful tool for quantifying carbon sequestration across different spatial scales and is thus fundamental for designing sustainable silvicultural practices and for carbon footprint compensation [4, 5].

Carbon assessments also provide baselines for forest management, support the development of nature-based management and underpin international initiatives such as REDD+ (Reducing Emissions from Deforestation and Forest Degradation [6, 7]). These maps are also essential for the bioeconomy, as well-managed forest resources are closely linked to regional well-being and the supply of ecosystem services like timber production, erosion control, pest control, energy, and water regulation [8]. Furthermore, high-resolution, locally validated biomass data have revealed positive relationships between biodiversity and carbon pools [9, 10]. In summary, accurate carbon mapping improves decision-making, supports sustainable forest management, conserves local biodiversity and optimizes carbon sequestration by reforestation [11, 12].

To estimate these stocks, methods range from traditional field surveys to advanced remote sensing [13]. Field measurements of tree height and diameter at breast height (dbh) are highly accurate but time-consuming, costly, and spatially limited [5]. Consequently, remote sensing has become essential for large-scale mapping. Satellite missions such as Sentinel and Landsat provide high-resolution multispectral imagery suitable for monitoring forest cover and estimating aboveground biomass [13]. More recently, Airborne Laser Scanning (Light Detection and Ranging, LiDAR) has revolutionized the field by delivering detailed three-dimensional information on forest structure, which is strongly linked to biomass and carbon stocks [14–17].

These data are often integrated with machine learning algorithms such as Random Forest (RF [18]) and Generalized Boosted Regression (Boosted Regression Tree, BRT [19]) to model non-linear relationships between remote sensing signals and forest attributes [20–22]. While most studies focus on aboveground biomass (AGB) and carbon stocks, belowground biomass (roots, BGB), litter, dead wood and soil carbon are also critical components of carbon pools and may require specific assessment approaches [23]. In Mediterranean forests, belowground biomass and litter together represent up to 61% of the aboveground stock [24]. Furthermore, forest canopy cover, summer precipitation and vegetation type have been identified as the main drivers of forest floor carbon stocks in these systems [23].

Carbon stocks have been studied on different levels, from a regional to a global scale [25] and in different biomes. A great effort has been made to estimate biomass in the tropics [26–28] but also in temperate forests [29, 30]. In the Mediterranean region, carbon mapping of forests has revealed a strong variation in carbon density among forest types driven by pronounced climatic gradients, seasonal aridity, and a long history of human use that has profoundly altered the forest structure [31]. These forests frequently occur as fragmented mosaics due to fine-scale land use variability [5, 13, 17, 31].

Similarly, carbon mapping on oceanic islands presents a unique context and substantial challenges. These territories are often characterized by extreme environmental heterogeneity, where climate, vegetation types, and land use change markedly over very short distances [32–34]. Island ecosystems are inherently fragile due to their isolation and limited physical space which shape the evolutionary trajectories of their biotas [35]. Moreover, island forests undergo greater proportional rates of change in composition and extent than mainland ecosystems, largely driven by alien invasive species, drought, and intensive and rapid land-use shifts linked to generally high population density [36, 37].

Complex topography and limited accessibility on oceanic islands constrain carbon mapping, making repeated ground inventories challenging and airborne laser scanning costly. These islands are widely recognized as biodiversity hotspots with exceptional levels of endemism [38], positioning them as key model systems for biodiversity conservation-oriented forest management and for evaluating the biodiversity-co-benefits of carbon-targeted initiatives [10]. Biodiversity-carbon relationships have been reported to depend on trophic levels and type of carbon storage compartment in subtropical forests [39]. High-resolution carbon mapping could therefore make a critical contribution to the conservation of endemic-rich island forest ecosystems. However, island-wide studies on carbon stocks and carbon mapping are limited [14, 40, 41]. Asner et al. [14], for example, produced the first carbon map of the Hawaiian archipelago, identifying canopy cover, mean annual precipitation and vegetation type as main predictors.

Stand-level biomass and carbon stocks have been assessed regionally in the Azores, distinguishing between natural forests, production forests and exotic woodlands [40, 42]. On Madeira, Massetti & Gil [41] calculated aboveground biomass carbon stocks using multispectral satellite data and available vegetation maps. In the Canary Islands, estimates of forest carbon stocks have mainly been carried out at the stand level based on data from the Spanish National Forest Inventory (NFI [43]) and species-specific allometric equation models [44–48]. However, no attempt has yet been made to map carbon stocks on the Canary Islands as a whole.

On the Canary Islands, three main forest types have been described and mapped [49]: thermophilous woodlands, laurel forest and pine forest. The first two forest types have been severely destroyed, degraded and damaged by human activities to the point that on some of the islands only very small remnants (< 5% of the potential area) remain [50–53]. Yet they are considered biodiversity hotspots and harbor high levels of taxonomic, phylogenetic and functional diversity [54]. This habitat loss entails an extinction debt, especially for range-restricted endemic species [51]. In contrast, Canary pine forests, although historically exploited for timber, have been extensively reforested, especially on Tenerife and Gran Canaria, and today occupy a large proportion of their potential distribution. High-resolution carbon assessment and mapping on the Canary Islands are therefore crucial to improve our understanding of ecosystem functioning and carbon fluxes, to inform forest management and conservation planning, to estimate carbon losses or gains due to land-use change, and to support accounting for CO_2_ emissions and removals in line with the regional goal of carbon neutrality [55].

The main objective of this study was to develop an integrated assessment of carbon stocks across all forested Canary Islands. First, stand-level carbon accumulation was estimated by calculating above- and belowground biomass tree by tree, using structural plot data from the Spanish NFI, which is regularly distributed across the archipelago, together with updated, species-specific allometric equations. In the second step, high-resolution modelling and mapping of carbon stocks were carried out by extrapolating plot-based estimates to the full spatial extent of current forest types, using LiDAR data, satellite imagery, and machine-learning algorithms. We identified the factors driving the spatial distribution of these carbon stocks. Furthermore, the performance of different statistical models was evaluated, and our predicted carbon density maps were compared to existing satellite-based biomass/carbon products. Finally, implications for future forest management, land-use planning, and the role of biodiversity in carbon storage were discussed.

Overall, the novelty of our island-specific approach to carbon stock and carbon density estimation, compared to previous studies, lies in several key aspects, including both above- and belowground carbon stocks, the use of an extensive set of plot-based field data for model calibration on individual islands, fine-scale interpolated climatic data, updated species-specific allometric equations for 50 tree species, a detailed forest-type classification and benchmarking against global biomass products.

## Methods

### Study area and vegetation classification

The Canary archipelago lies 96 km off the arid northwest coast of Africa and forms part of the Macaronesian biogeographical region, including the Azores, Madeira and Cabo Verde [56]. Island age decreases from east to west across the Canarian Archipelago with Fuerteventura being the oldest (21 Ma) and El Hierro the youngest island (1.1 Ma). However, all islands except La Gomera remain volcanically active with eruptions during the last few thousand years. The Canaries are characterized by a Mediterranean-type climate and are renowned for their remarkable ecosystem diversity. Steep climatic gradients occur especially on the northern, more humid slopes of the five higher (> 1450 m) islands (Tenerife (T), Gran Canaria (C), La Gomera (G), La Palma (P), and El Hierro (H)), whereas the two eastern islands, Lanzarote (L) and Fuerteventura (F), are lower in elevation and show less environmental variation.

The distribution of the three major native forest ecosystems among the Canary Islands is as follows (Fig. S1) [49]: thermophilous woodlands currently present on five islands (T, C, G, P, H), potentially on seven islands (plus L and F); laurel and pine forests on five islands (T, C, G, P, H). Representative species of the thermophilous woodlands still grow on the eastern islands (L, F) but no longer form forest communities due to human disturbances [53].

These main forest ecosystems have been subdivided into phytosociological units and mapped for the whole archipelago [49]. We reclassified the phytosociological associations into broader categories, distinguishing 18 forest types, including native forest formations as well as plantations of exotic tree species, such as Monterrey pine (*Pinus radiata*), *Eucalyptus* species and chestnut (*Castanea sativa*). Using a Geographic Information System [57], the original vegetation map of the Canary Islands [49] was modified and used both as a predictor for the spatial modelling of aboveground and belowground carbon densities and to calculate the overall carbon stocks for each forest type and on each island. In this framework, thermophilous woodlands encompass open woodlands dominated by small trees such as *Juniperus turbinata* ssp. *canariensis* (Canarian juniper), *Olea cerasiformis* (Canarian olive tree), and *Phoenix canariensis* (Canarian palm). Laurel forest was divided into dry (LS), typical (L) and humid laurel forest (LH, see precipitation characteristics for laurel and pine forest types in Table S1). Furthermore, natural formations of *Pinus canariensis* were differentiated into humid (PH), typical (PT), dry (PS) and high mountain (PAM) pine forests, and plantations of the Canarian pine were separated by stand density (high-RDA, medium-RDM and low-RDB). Additionally, we distinguished a forest formed by *Morella faya* and *Erica arborea*, plantations of exotic species such as *Pinus radiata* (PPR), *Eucalyptus* spp. (EUC) and *Castanea sativa* (C), as well as plantations of other exotic conifers and broad-leaved species (PCF).

### Forest plot data

Data for estimating above- and belowground biomass and derived carbon densities were obtained from the Spanish National Forest Inventory (NFI). The NFI consists of a network of permanent circular plots used to monitor forest conditions, with measurements repeated every ten years throughout the Spanish territory. In the Canary Islands, sampling follows a systematic 500 × 500 m UTM grid. Grid cells are included when forest cover exceeds the 10% threshold based on aerial photo interpretation. During the most recent survey in 2017 (NFI 4; Fig. S2 [43]), 1,834 plots were sampled in the archipelago: 1,347 in the province of Santa Cruz de Tenerife (T, P, G, H) and 487 on the island of Gran Canaria which belongs to the province of Las Palmas together with Fuerteventura and Lanzarote. The very few NFI plots in Fuerteventura (10–20) do not represent forest ecosystems and are not included in the modelling approach here. The NFI plots are circular with four nested concentric subplots with the following thresholds for sampling: (i) Trees with 7.5 cm ≤ dbh < 12.5 cm were measured in the 5 m subplot. (ii) Trees with 12.5 cm ≤ dbh < 22.5 cm were measured in samples of 10-m radii. (iii) Trees with 22.5 cm ≤ dbh < 42.5 cm were measured within the 15-m-radius subplot, and (iv) Trees with 42.5 cm ≥ dbh were assessed in the last concentric 25 m subplot. The inventory provides detailed information on plot location (geographical coordinates, elevation, substrate type, etc.) and on stand structure and composition (dbh, height, stand density, etc.).

Aboveground (ACD), belowground (BCD) and total (TCD) carbon densities were calculated at individual tree and plot level (37,216 trees, 1,674 plots) using the most up-to-date species-specific allometric equations for 50 tree species (see detailed information in Table S2) [46, 58–60]. In the case of the laurel forest, allometric equations for the four most abundant tree species (*Erica arborea*, *Ilex canariensis*, *Laurus novocanariensis*, *Morella* (*Myrica*) *faya*) were available, while a generic equation based on these four species was constructed for the ten remaining less abundant tree species [60, 61]. Where no species-specific equation was available, equations for congeners from the Iberian Peninsula with similar growth forms and environments were applied (Table S2). Tree regeneration, including all juvenile individuals with 2.5 cm ≤ dbh < 7.5 cm and height > 130 cm, was incorporated by applying the same allometric equations developed for adults. All tree allometries were derived from power-law equations of the form BM = a(dbh²xh)^b, where diameter at breast height (dbh, cm) and tree height (h, m) were used as predictor variables, following previously published allometric databases [60, 61]. Understory shrub biomass was estimated using allometric equations for ecologically similar shrub species from the Iberian Peninsula, based on community cover (%) and mean height (m) (see detailed information in Table S3) [62, 63]. Species-specific allometric equations were developed for several abundant endemic shrub species, relating biovolume (cylinder defined by mean canopy diameter and maximum height) to dry biomass. Root biomass was estimated using root/shoot ratios and existing allometric equations for the tree and understory shrub species of the Canary Islands and the Iberian Peninsula (Table S4, S5) [58, 59, 61]. Carbon content of dry above- and belowground biomass was taken from Montero et al. [62, 63] for trees and shrubs, respectively.

### Predictor variables

Thirty-two potential predictor variables for estimating TCD were compiled from open-access sources covering the entire archipelago (Table 1). Geographic predictors comprised UTM X and Y coordinates (UTM, Universal Transverse Mercator) and a third-order trend surface generated with a linear model and predicted across each island [64]. Terrain variables (elevation, slope, aspect) were derived from a high-resolution digital elevation model (DEM) using the “terra” package in R [65]. The DEM layer with a spatial resolution of 5 × 5 m was downloaded from the National Geographic Institute of Spain and is based on LiDAR flights with a resolution of 0.5 points/m² conducted in 2009 as part of the first coverage of the National Aerial Orthophotography Plan (PNOA). Climate variables were obtained from the recent high-resolution CanaryClim dataset (100 × 100 m pixel size) [66]. Vegetation structural variables such as tree volume (timber volume over bark), basal area, maximum tree height, and canopy cover were derived from airborne laser scanning (ALS) by Guerra-Hernández et al. [65] and provided by the Spanish Ministry for Ecological Transition and the Demographic Challenge. The authors combined, for the first time, an Area-Based Approach (ABA) with ALS techniques across the Canary Islands, by integrating field measurements obtained from the NFI4 plots in 2017 with low-density (0.5 pulses/m2) airborne LiDAR data collected in 2015–2016 and freely available through the Land Survey Institute (*Plan Nacional de Ortofotografía Aérea*, PNOA). The generated height and canopy LiDAR metrics served as independent variables in linear regression models calibrated to predict essential forest structural variables which were then spatially expanded across defined vegetation strata through raster-based mapping with a spatial resolution of 25 m. Only NFI4 plots with high-precision geo-localization using global navigation satellite systems (GNSS) were used for modelling to avoid the co-registration error associated with ALS-based forest inventory [16, 67]. The temporal mismatch between NFI measurements and ALS data is negligible.

Finally, Sentinel-2A spectral bands were downloaded from the Copernicus Browser [68] for cloud-free dates in January and April 2020 (P, H) and 2021 (T, C, G), including bands 02–04 (visible: blue, green, red), 05–07 (red edge), 08–09 (near-infrared, water vapour) and 11–12 (shortwave infrared, SWIR1, SWIR2). Dates were selected to avoid the effects of wildfires that occurred in Canary pine forests and *Erica-Morella* forests in 2021, 2022 and 2023. Vegetation indices were also calculated to enhance vegetation–soil discrimination: the Normalized Difference Moisture Index (NDMI = (B8A – B11)/(B8A + B11)), reflecting vegetation and soil moisture; the Normalized Difference Vegetation Index (NDVI = (B8 – B4)/(B8 + B4)), representing vegetation greenness; and the Normalized Difference Water Index (NDWI = (B3 – B8)/(B3 + B8)), reflecting vegetation water content. Since source data vary in spatial resolution (5–100 m, Table 1), predictor layers were resampled to 50 × 50 m resolution, a spatial grain recommended for carbon mapping and matching approximately the NFI plot size of 1,963.5 m^2^ (plots of 25 m radius) [69]. To facilitate ecological interpretation, a correlation matrix of the predictors was constructed (Fig. S3).

**Table 1:** Characteristics of 32 explanatory variables used for predictive modelling of carbon density in forests of the Canary Islands

Category	Variable	Code	Source
Geographical	X coordinate	XWGS84	
	Y coordinate	YWGS84	
	Third order trend surface	trend	
Terrain	Elevation	dem	
	Exposition	exp	
	Slope	slope	
Climatic	Mean annual temperature	pmed	Patiño et al. (2013)
	Temperature annual range	tann	
	Temperature seasonality	tseas	
	Mean annual precipitation	pmed	
	Precipitation of the driest quarter	pdry	
	Precipitation of the wettest quarter	pwet	
	Air humidity	hum	
Vegetation	Simplified digital vegetation map of 18 main forest types	vegtype	del Arco et al. (2010)
Airborne laser scanning (ALS)	Mean crown height	alt	Guerra-Hernández et al. (2019)
	Canopy cover	cover	
	Stem density	dens	
	Volume of timber over bark	vol	
Sentinel spectral bands	B2 blue	b2	Sentinel 2 A
	B3 green	b3	
	B4 red	b4	
	B5 near infrared	b5	
	B6 near infrared	b6	
	B7 near infrared	b7	
	B8 vegetation red	b8	
	B9 water vapour	b9	
	B11 short wave infra-red, SWIR1	b11	
	B12 short wave infra-red, SWIR2	b12	
	Moisture index (NDMI)	moist	
	Normalized Difference Vegetation Index	NDVI_spr	
	Normalized Difference Vegetation Index	NDVI_win	
	Normalized Difference Water Index	NDWI	

### Spatial modelling

To model aboveground, belowground and total biomass carbon densities (ACD, BCD and TCD), we employed Boosted Regression Trees (BRT) and Random Forest (RF). BRT, also known as Generalized Boosted Regression models (GBMs), is a tree-based machine learning technique for building an ensemble of regression trees in a sequential manner. The initial tree is fitted to the training data, while subsequent trees are fitted to the residuals of the preceding tree. Each tree’s prediction is weighted, and the residuals at each step are calculated as the difference between the current residuals and the weighted predictions of the new tree. The final BRT prediction is the sum of the weighted outputs from all trees in the ensemble [70, 71]. BRT is well-suited for modelling non-linear relationships and variable interactions, and it is robust across various data distributions, including Gaussian, proportional (continuous), and count (Poisson) data. Modelling was performed using the “gbm” R package [19] and “spm2” [71].

We tuned our BRT models by optimizing key hyperparameters: interaction.depth, train.fraction, and n.minobsinnode. Model tuning aimed to maximize explained variance. Variable selection was conducted using the functions stepgbmRVI and steprfAVIPredictors from the “stepgbm” and “steprf” R packages, respectively. This process enabled us to identify an optimal subset of predictors based on their predictive contributions.

We further examined the relative influence of predictors and their partial responses using the partialResponse function from the “predicts” package [72], providing insights into variable effects on carbon density prediction. Additionally, we applied the RF algorithm using the “randomForest” package [73]. RF is an ensemble method that constructs multiple unpruned regression or classification trees from bootstrapped samples of the training data. At each node split, a random subset of predictors is considered. For regression, predictions are averaged across all trees, while for classification, a majority vote is used [18]. When all predictors are considered at each split, RF reduces to bagging [70]. Model tuning involved optimizing mtry and ntree. The tuneRF function was used to determine the optimal mtry value, with ntree fixed at 500, based on minimizing out-of-bag (OOB) error. Feature selection for RF was performed using the Boruta algorithm via the “Boruta” package [74, 75], identifying all relevant predictors from the original dataset.

To compare models’ performance, we implemented a manual tenfold cross-validation using the folds function from the “predicts” package. Model evaluation metrics included the coefficient of determination (R^2^), Root Mean Squared Error (RMSE) and Mean Absolute Error (MAE). Each metric was computed using the “metrica” R package [76]. Finally, spatial predictions were generated using the predict function from the “terra” package. We ran all island models (BRT and RF) with and without LiDAR variables to evaluate the overall importance of this group of predictors for model outcomes.

Above-, belowground and total carbon stocks (ACS, BCS, TCS) were calculated by intersecting the corresponding carbon density raster maps with the forest map aggregating all pixel values for each forest type across islands.

Uncertainty in our predictions was assessed using a bootstrapping approach (*n* = 100; [77]. At each bootstrap iteration, models were refit using a random subset comprising 75% of the data, with a different random seed used for each iteration. Prediction uncertainty was quantified at the pixel level as the standard deviation of predictions across all iterations for each island. This procedure was applied separately to the best random forest or GBM model, resulting in 10 uncertainty maps: five islands x best modelling approach x 2 carbon measures.

Generalized additive models (GAMs) were performed to analyze and visualize the influence of moisture and temperature on TCD at the archipelago level using all available NFI plots and applying the “gam” function from the “mcgViz” package [78].

### Comparison with existing biomass products

To benchmark available biomass products and compare them with our predicted above-ground carbon density maps, we used two independent biomass sources: ESA CCI Above-Ground Biomass maps (AGBD; [79]) and Global Ecosystem Dynamics Investigation (GEDI) Level 4 A (L4A) footprints [80].

First, we downloaded the ESA CCI Above-Ground Biomass raster maps for 2022 (the nearest year available to 2020 and 2021) at 100 m spatial resolution using Google Earth Engine [81]. This dataset provides estimates of forest above-ground biomass derived from multiple Earth observation sources, including, depending on the year, data from the Copernicus Sentinel-1 mission, Envisat ASAR, and JAXA ALOS-1 and ALOS-2, together with ancillary Earth observation information. The product was generated by the Biomass CCI team within the European Space Agency Climate Change Initiative program.

Second, we retrieved GEDI L4A footprints in point format at 25 m resolution for 2020 and 2021 using the QGIS plugin GEDI Metrics [82]. We acknowledge that this step may have some reproducibility limitations because it depends on the plugin workflow and processing configuration.

GEDI Metrics enables the integration of the GEDI processing chain within a GIS environment, including variable extraction, quality filtering, spatial subsetting, and product integration. Before using the GEDI L4A data, we applied several quality-control filters: we removed degraded footprints, retained only footprints with sensitivity values greater than 0.9, kept only land-surface observations present across all required products, and selected only full-power beams (BEAM0101, BEAM0110, BEAM1000, and BEAM1011) as recommended for dense forest conditions.

Finally, we compared our predicted above-ground carbon density maps with both external biomass products (converted into aboveground carbon applying the conversion factor 0.5). For comparison with the ESA CCI AGBD rasters, our maps were aggregated to 100 m resolution, and only spatially coincident pixels were retained. For comparison with GEDI L4A, values from our maps were extracted at GEDI footprint locations retaining the mean value of the AGBD when the 50 m resolution grid contained more than one footprint, using our maps at 50 m resolution.

## Results

### Carbon stocks and densities

**Fig. 1:**
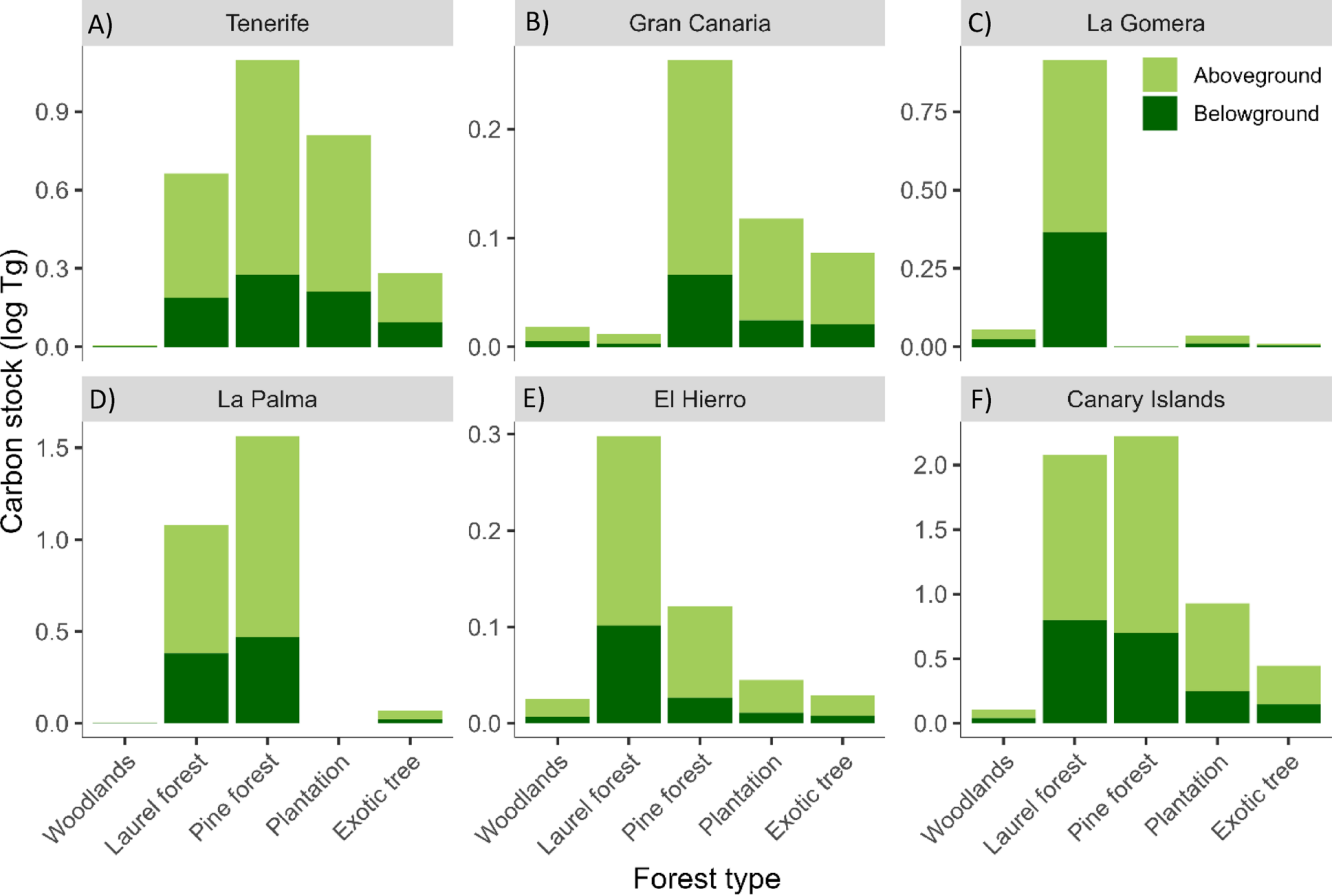
Total (aboveground and belowground biomass) carbon stocks (Tg) for different forest groups and island applying best fitting model Boosted Random Tree (BRT) or Random Forest (RF). Woodlands: thermophilous woodlands (S, ACL, P), Laurel forest: laurel forest and *Erica-Morella* forests (LH, L, LS, FB), Pine forest: Canarian pine forest (PH, PT, PS, PAM), Plantation: Plantations of Canary pine (RDA, RDM, RDB), Exotic tree: Plantation of exotic tree species (PCF, PPR, EUC, C). See Fig. 3 legend for abbreviations of forest types. Observe that the scales used in the different islands are variable. (**A**) Tenerife, **B**) Gran Canaria, **C**) La Gomera, **D**) La Palma and **E**) El Hierro, **F**) Canary Islands

**Fig. 2:**
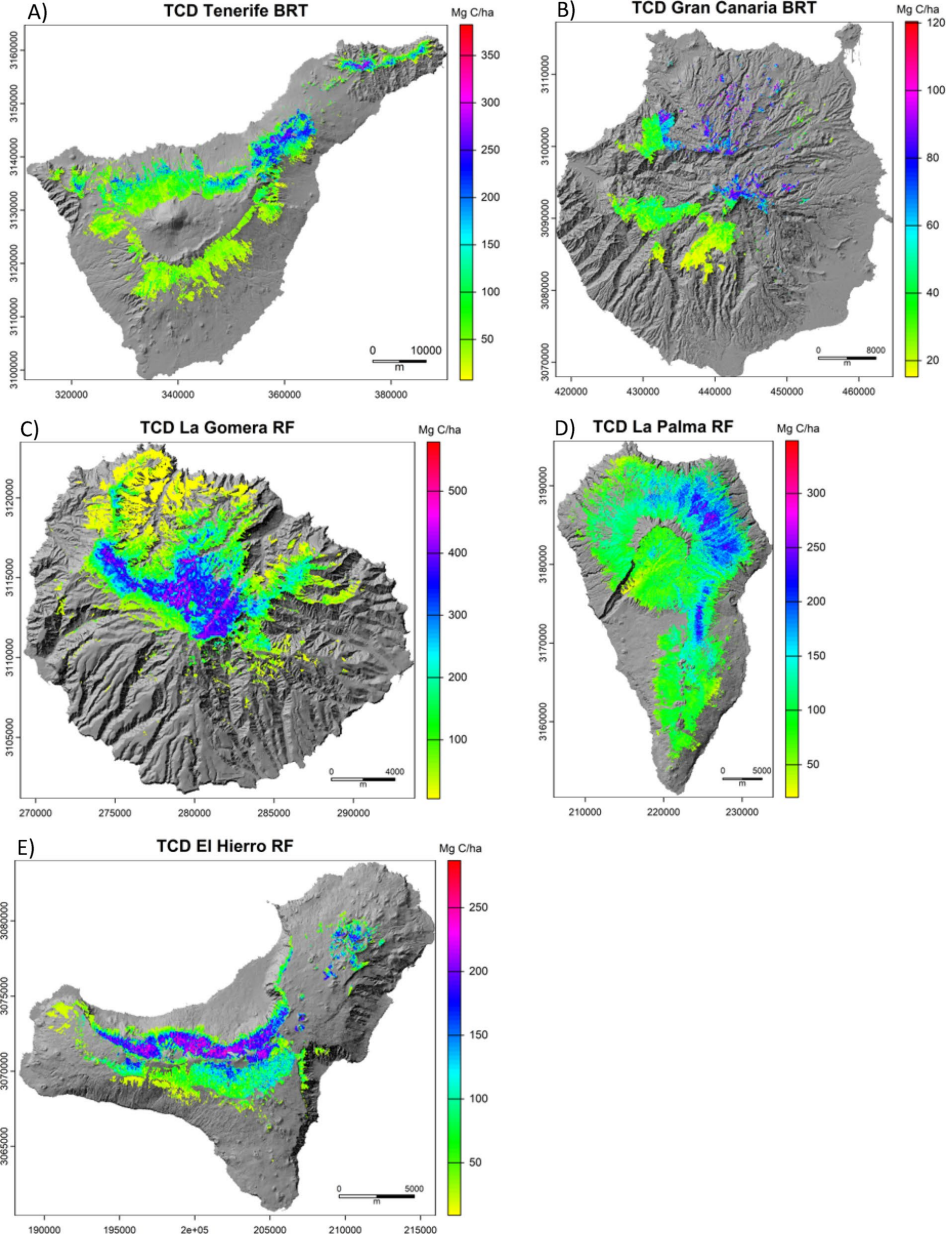
Spatial distribution of forest total (aboveground and belowground) carbon density (TCD, Mg C ha^− 1^) for each Canarian island at 50-m mapping resolution. Best models used to calculate total carbon stocks included Boosted Regression Tree (BRT) and Random Forest (RF). Color ramp values are adapted to the maximum and minimum values of each island. (**A**) Tenerife, **B**) Gran Canaria, **C**) La Gomera, **D**) La Palma and **E**) El Hierro

On the entire Canarian archipelago, forests currently occupy a total area of 112,735 ha and store 10.26 Tg (million metric tons) of carbon (TCS) (Figs. 1 and 2; Table 2), of which 7.55 Tg (73.6%) correspond to aboveground and 2.72 Tg (26.4%, ACS) to belowground biomass (BCS). La Palma comprises 32.2% of the total Canarian forest cover and 40% (4.14 Tg) of the total Canarian forest carbon stock (TCS), followed by Tenerife with 42.2% forest cover and 37.5% (3.77 Tg) of the total carbon stock (Fig. 1). La Gomera accounts for 8.9% of the total forest cover and 12.4% (1.28 Tg) of the forest carbon pool. The two remaining islands (C, H) occupy 11.8% and 4.9%, respectively, of the forested area and each hold about 5% (0.53 Tg) of the total carbon accumulated on the archipelago.

**Table 2:** Forest cover and carbon stocks (aboveground and belowground) for each forest type and island applying best fitting model Boosted Regression Tree (BRT) or Random Forest (RF)

Island	Forest type	Cover (ha)	Carbon stock (Tg)		Total	Total%
			Aboveground	Belowground		
Tenerife		47625.7	2.904	0.864	3.767	
	Thermo	155.8	0.003	0.001	0.005	0.1
	Laurel	8620.6	0.606	0.208	0.814	21.6
	Pine	25331.1	1.272	0.319	1.590	42.2
	Plant	10660.4	0.817	0.236	1.054	28.0
	Exotic	2857.8	0.206	0.099	0.305	8.1
Gran Canaria		13328.2	0.405	0.123	0.528	
	Thermo	507	0.013	0.006	0.019	3.5
	Laurel	207.5	0.009	0.003	0.012	2.2
	Pine	8672	0.218	0.069	0.287	54.2
	Plant	2294.1	0.098	0.025	0.123	23.2
	Exotic	1647.6	0.067	0.021	0.089	16.8
La Gomera		10014.5	0.792	0.484	1.276	
	Thermo	2583.1	0.030	0.026	0.056	4.4
	Laurel	6507.4	0.731	0.442	1.173	91.9
	Pine	42	0.001	0.001	0.001	0.1
	Plant	554	0.024	0.011	0.036	2.8
	Exotic	328	0.006	0.004	0.010	0.8
La Palma		36277.0	3.053	1.086	4.140	
	Thermo	69.6	0.003	0.001	0.004	0.1
	Laurel	11099.3	1.013	0.463	1.476	35.7
	Pine	24497.3	1.988	0.599	2.587	62.5
	Plant	0	0.000	0.000	0.000	0.0
	Exotic	610.8	0.049	0.023	0.072	1.7
El Hierro		5490.5	0.391	0.160	0.550	
	Thermo	822.5	0.018	0.007	0.025	4.6
	Laurel	2315.8	0.217	0.107	0.324	58.8
	Pine	1448.8	0.100	0.027	0.127	23.0
	Plant	543.8	0.035	0.011	0.046	8.3
	Exotic	359.6	0.021	0.008	0.029	5.3
Canary Islands		112734.9	7.545	2.716	10.261	
	Thermo	4137.8	0.068	0.041	0.109	1.1
	Laurel	28750.3	2.575	1.223	3.798	37.0
	Pine	59991.1	3.579	1.013	4.592	44.8
	Plant	14052.1	0.974	0.283	1.257	12.3
	Exotic	5803.6	0.349	0.155	0.505	4.9

**Fig. 3:**
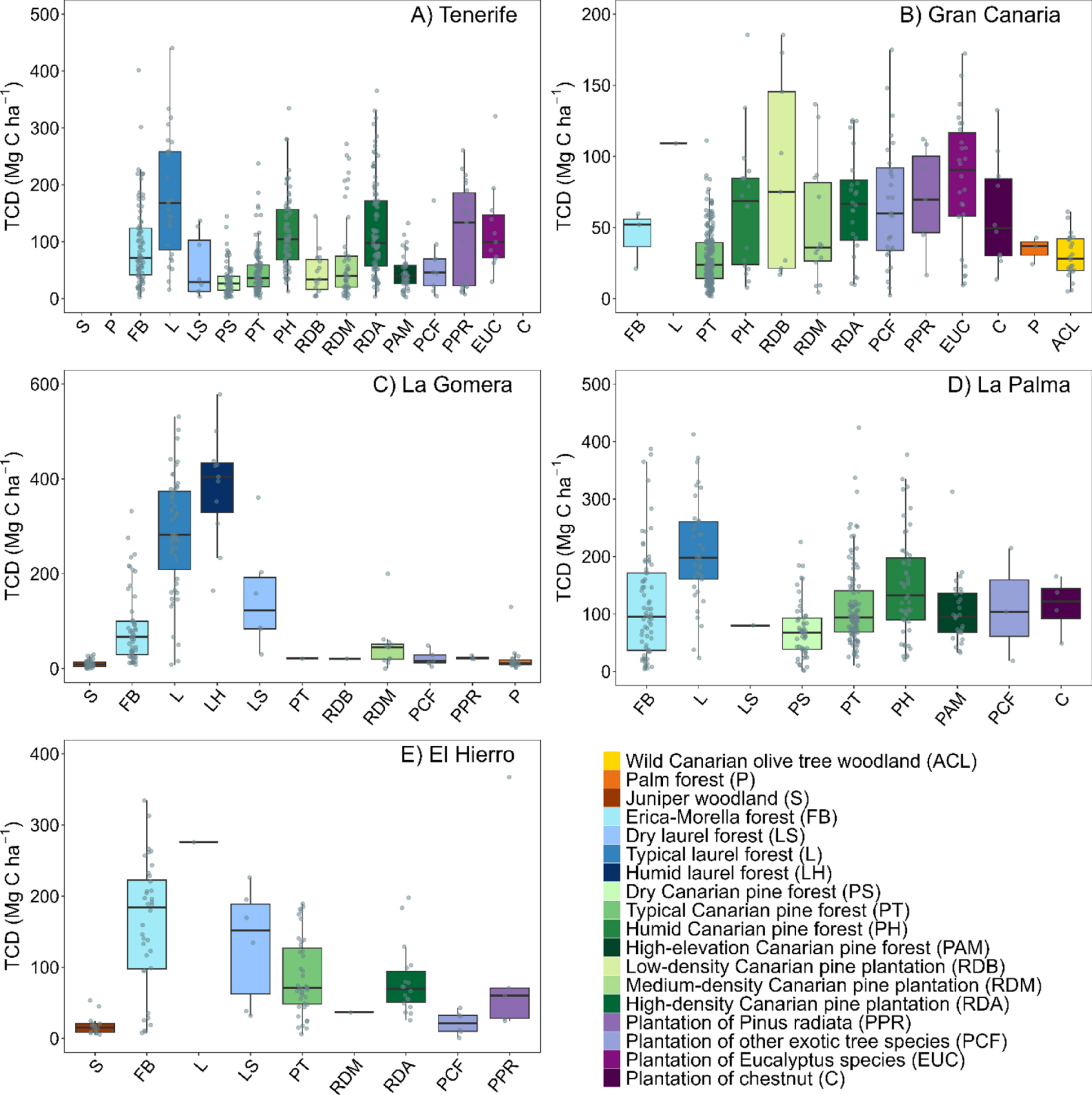
Total (aboveground and belowground) carbon density (TCD, Mg C ha^− 1^) for each forest type and island. Note that different scales are used for each island. (**A**) Tenerife, **B**) Gran Canaria, **C**) La Gomera, **D**) La Palma and **E**) El Hierro

Thermo: thermophilous woodlands (S, ACL, P), Laurel: laurel forest and heathland (LH, L, LS, FB), Pine: Canarian pine forest (PH, PT, PS, PAM), Plant: Plantations of Canary pine (RDA, RDM, RDB), Exotic: Plantation of exotic tree species (PCF, PPR, EUC, C). See Fig. 3 legend for abbreviations of forest types

Forest TCS values varied widely not only by island, but also by forest type (Fig. S4, Table S6). More than half of the total carbon is stored in Canarian pine forests (57.1%, of which 44.8% in natural stands and 12.3% in pine plantations), followed by evergreen broadleaved forests (laurel forest and *Erica*-*Morella* forest) which together contain 37% of the archipelago carbon pool. Exotic tree plantations (mainly *Pinus radiata*,* Eucalyptus globulus,* and *Castanea sativa*) only account for about 5% of forest TCS and cover, while the current carbon stored by thermophilous woodlands showed the lowest values (1.1% C, 3.7% cover) despite the potential distribution they had in the past [49–51]. ACD maps showed high similarities with those of TCD with small differences related to root/shoot ratios of dominant tree species (Fig. S4).

Reflecting the spatial distribution of forests within the archipelago (Fig. S2), the partitioning of TCS among forest types varies substantially between islands (Fig. 3, Table S6). On La Gomera, laurel forest exclusively accounts for 91.1% of the carbon pool, showing a typically high proportion of the belowground carbon stock (BCS 40%), while laurel and pine forests dominate carbon stocks on La Palma (35.7%, 62.5%, respectively). On Gran Canaria, natural and planted Canarian pine forests store most of the carbon (54.2%, 23.2%, respectively), exhibiting a lower BCS proportion (22%), and with exotic tree plantations representing the third most important carbon stock (16.8%). Tenerife and El Hierro display a more balanced carbon partitioning (Fig. 1).

The highest forest TCDs were found on La Gomera for humid and typical laurel forests, reaching mean values of 413.2 ± 149.5 and 285 ± 122.1 Mg C ha^− 1^, respectively, followed by humid and typical pine forests on La Palma, supporting 148.5 ± 87.8 and 113 ± 70.3 Mg C ha^− 1^, respectively (Figs. 2 and 3, Fig. S4, Table S7). The absolute maximum TCD values per island were 730 Mg C ha^− 1^ (490 Mg C ha^− 1^ ACD, 230 Mg C ha^− 1^ BCD, La Gomera, Laurel forest), 440 Mg ha^− 1^ (305 Mg C ha^− 1^ ACD, 135 Mg C ha^− 1^ BCD Tenerife, Laurel forest) and 424 Mg C ha^− 1^ (334 Mg C ha^− 1^ ACD, 90 Mg C ha^− 1^ BCD La Palma, Pine forest, Fig. 3, Fig. S5). Lowest mean TCD values between 10 and 70 Mg C ha^− 1^ were recorded for the variants of thermophilous woodlands, while TCDs for exotic tree plantations were generally high (100–120 Mg C ha^− 1^), depending on species and stand densities. We observed not only high inter-community but also intra-community variation in TCDs related to differences in species composition, stand structure and densities. For instance, NFI plots of widely abundant *Erica-Morella* forests on all five islands revealed TCDs ranging from 20 to 250 Mg C ha^− 1^. The same pattern was detected for laurel forest stands (30–730 Mg C ha^− 1^).

### Model comparison

**Fig. 4:**
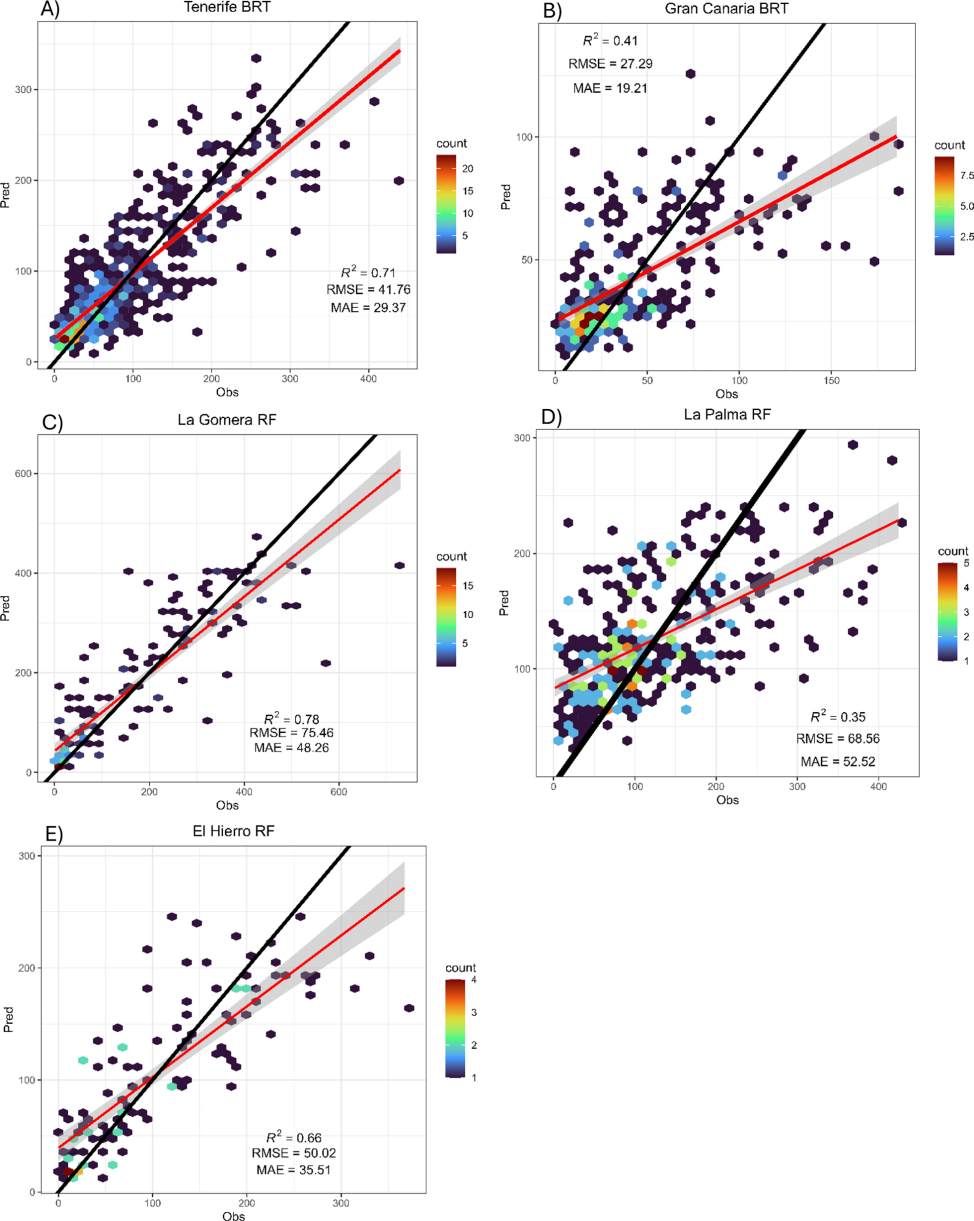
Relationships between predicted and observed total carbon densities (above and belowground biomass) across National Forest Inventory sites on each of the Canary Islands. Panels represent best fits of Boosted Regression Tree (BRT) or Random Forest (RF) models to calculate total carbon stocks for the entire forested area of the islands. Grey shaded areas represent variability in predictions from models with different hyperparameter configurations. Red lines depict the linear regressions between observed and predicted values for models. Solid black lines represent 1:1 linear fit. Summary statistics of model evaluation show metrics including the coefficient of determination (R²), Root Mean Squared Error (RMSE) and Mean Absolute Error (MAE). Color ramp shows the number of plots with similar values. Models were validated using 10-fold cross-validation. Observe that the scales used in the different islands are variable. (**A**) Tenerife, **B**) Gran Canaria, **C**) La Gomera, **D**) La Palma and **E**) El Hierro

Comparison of Boosted Regression Tree (BRT) and Random Forest (RF) models unveiled very similar performances (Table 3), and the selection of the best-fitting models was based on cross-validation statistics (R^2^, RMSE, MAE) and changed depending on the island. Therefore, calculations of TCS extracted from extrapolated maps differed only slightly between model types (2–4%, Table S7) with slightly higher stocks for RF models. Evaluation of best TCD models based on field data in El Hierro, Tenerife and La Gomera indicated overall good fits (R^2^ = 0.66, 0.71, 0.78) and high accuracy with RMSE of 9.7%, 9.5%, 10.4%, respectively (Fig. 4; Table 3, Table S8). Predictive models for Gran Canaria and La Palma explained 41% and 42%, respectively, of the variability in observed TCD and showed lower accuracy levels (14.8%, 16.3%, respectively). Relationships between predicted and observed values for best-fitted models by island are presented in Fig. 4. Deviations of the regression line from the 1:1 fit line revealed a general overestimation of low and an underestimation of high TCD values. Models with lower R^2^ showed a higher bias. These biases can also be noticed in the maps of estimated uncertainty indicating largest uncertainties of TCD in forests with very high biomass content, with much lower uncertainties in low biomass conditions (Fig. 4, Fig. S6 and S7).

**Table 3:** Results of validation of predictive models estimating total forest carbon densities on the five Canary Islands

	Model									
	BRT					RF				
Island	R^2^	RMSE	RMSE%	MAE	n	R^2^	RMSE	RMSE%	MAE	n
Tenerife	0.71	41.76	9.53	29.37	524	0.70	42.60	9.72	30.47	524
Gran Canaria	0.41	27.29	14.83	19.21	289	0.38	28.07	15.26	19.07	289
La Gomera	0.76	78.21	10.74	50.68	153	0.78	75.46	10.37	48.26	153
La Palma	0.42	65.7	15.55	47.67	309	0.35	68.58	16.34	52.52	309
El Hierro	0.65	49.51	13.49	35.57	116	0.66	50.02	13.62	35.51	116

Performance metrics for Boosted Regression Tree (BRT) or Random Forest (RF) models based on NFI plots (number of plot = n) are shown including the coefficient of determination (R²). Root Mean Squared Error (RMSE). Normalized RMSE% by range of observed values and Mean Absolute Error (MAE). Models were validated using 10-fold cross-validation

### Relative importance of predictor variables

**Fig. 5:**
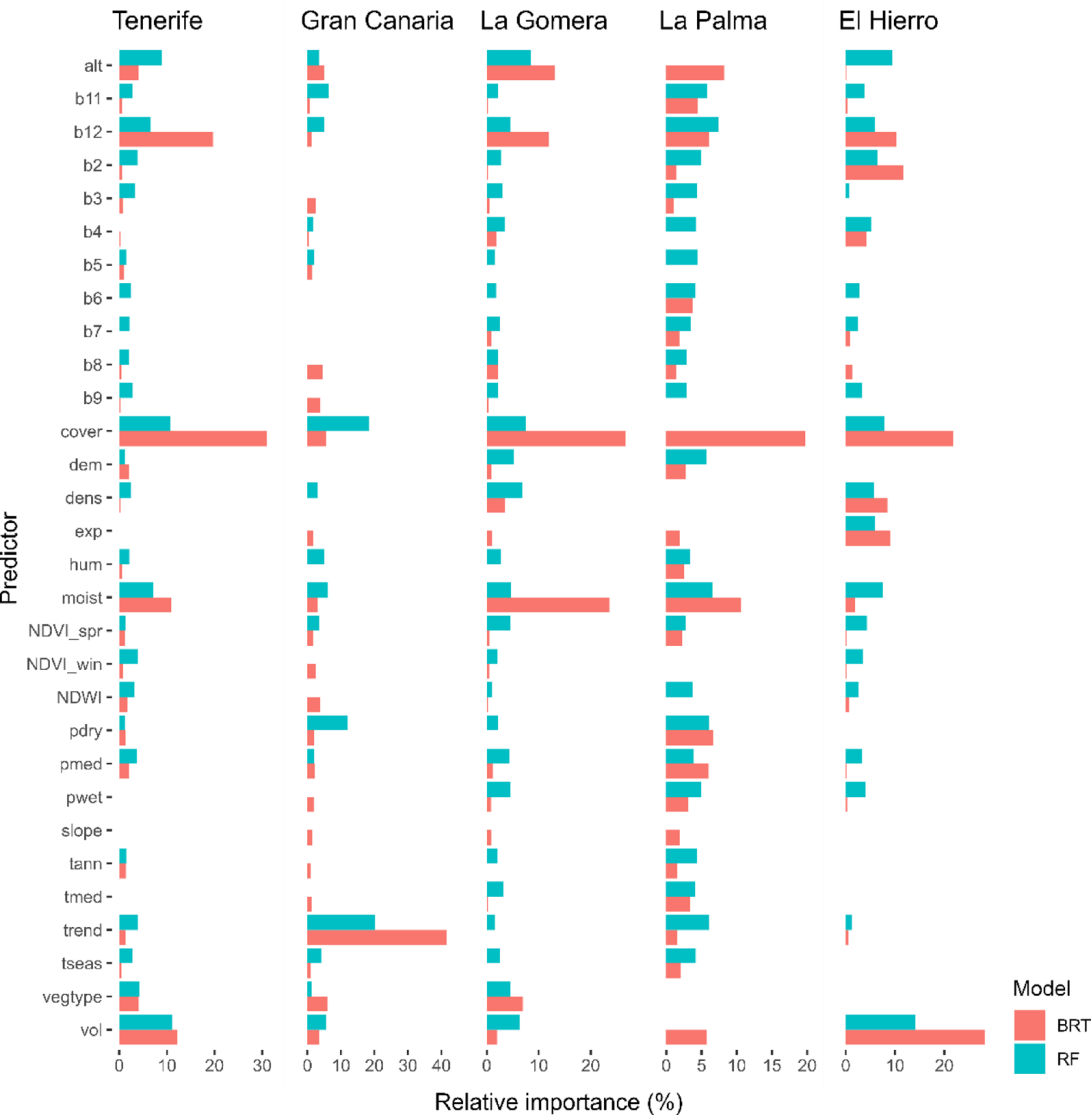
Relative importance (%) of predictors in models estimating total carbon densities (above and belowground biomass) across National Forest Inventory sites for five of the Canary Islands. Models represent best fits of Boosted Regression Tree (BRT) or Random Forest (RF) models. Abbreviations of predictors follow Table 1. Importance value in BRT models measures the relative contribution of each predictor variable to reducing prediction error across all trees and are scaled up to 100. The importance value in RF reflects the increase in Mean Squared Error (%) compared to the mean importance. We rescaled the RF importance value to a scale 0–100 for comparison with the BRT value

**Fig. 6:**
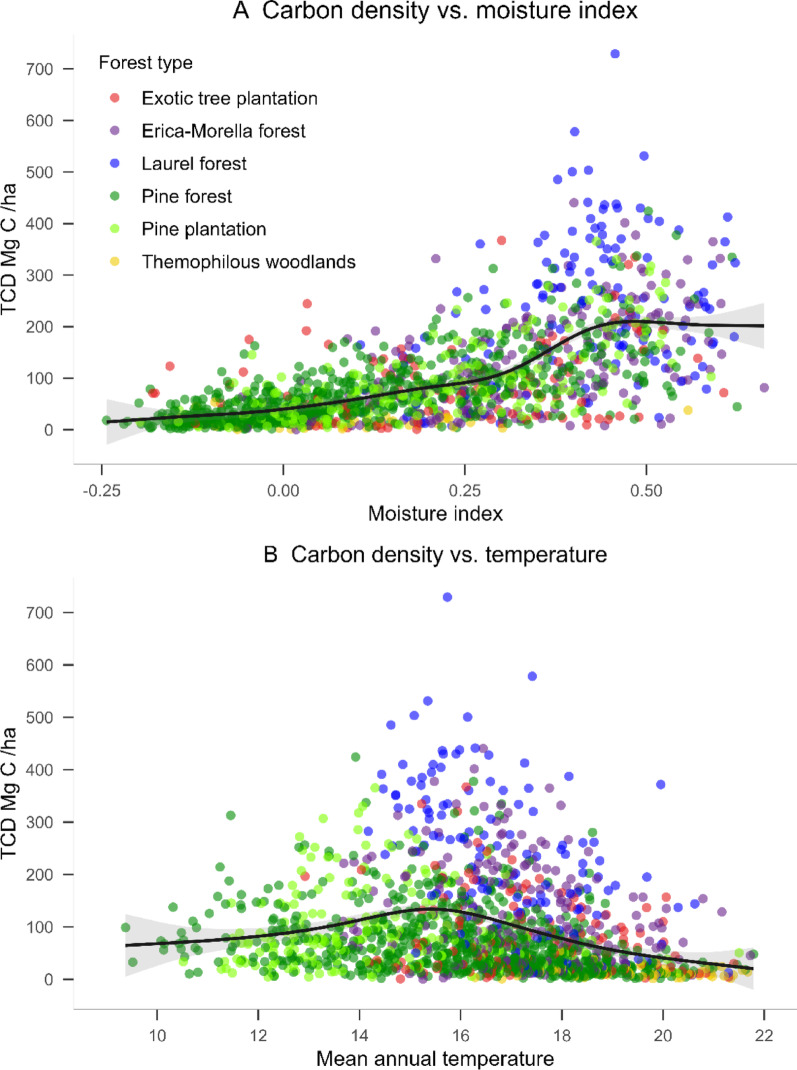
Relationship between carbon density and moisture index (A) and between carbon density and mean annual temperature (^o^ C) for National Forest Inventory plots on the Canary Islands (*n* = 1391) applying generalized additive models (GAMs). The Moisture Index (NDMI = (B8A – B11)/(B8A + B11)) was derived from Sentinel-2 A spectral bands and is reflecting vegetation water content

The correlation matrix of predictor variables (Fig. S3) revealed several associations between the TCD covariates. Most of the satellite spectral bands and derived indices exhibited positive correlations with precipitation variables, reflecting the availability of water resources. Many spectral bands as well as LiDAR derived variables (tree volume, cover and height) showed similar patterns and were positively correlated with each other (b2-b5, b6-b9, b11-b12, NDVI, NDWI, NDMI). The six predictors for TCD models with the highest relative importance values considering all five island models were three LiDAR-derived variables (tree volume, canopy cover and height), two satellite derived variables (moisture index, Sentinel-band b12) and forest type (Fig. 5). Results indicated that tree canopy cover was the principal driver of spatial variation in forest TCS throughout the Canarian archipelago, accounting for 20–31% of the variance explained by the model, followed by tree volume, both variables directly related to stand biomass. Moisture had a positive effect on carbon storage, whereas the Sentinel-band b12 was positively correlated to hydric stress and, consequently, negatively related to biomass and C densities. Despite this general pattern, we observed large differences in the variable ranking according to island and the type of model applied (BRT, RF). Spatial trend surface was, for example, the best predictor for TCD on Gran Canaria, while this variable had low importance on the other islands. On La Palma, where we ran the RF model without LiDAR variables due to a mismatch between these variables and the vegetation map, climatic variables were revealed to be more important. When LiDAR-derived variables were also removed from the other island models, climatic variables increased their importance values, while predictive power and accuracy of the models slightly decreased (Table S9, S10). Terrain variables (elevation, slope and exposition) generally showed low influence on TCD. Although co-variation among explanatory factors exists, predictor rankings include this effect by indicating the additional effect of each variable on TCD variation. GAM models showed a strong influence of moisture, but not of mean annual temperature on TCDs when all NFI plots on the five islands were analyzed (Fig. 6).

### Comparison with other biomass products

**Fig. 7:**
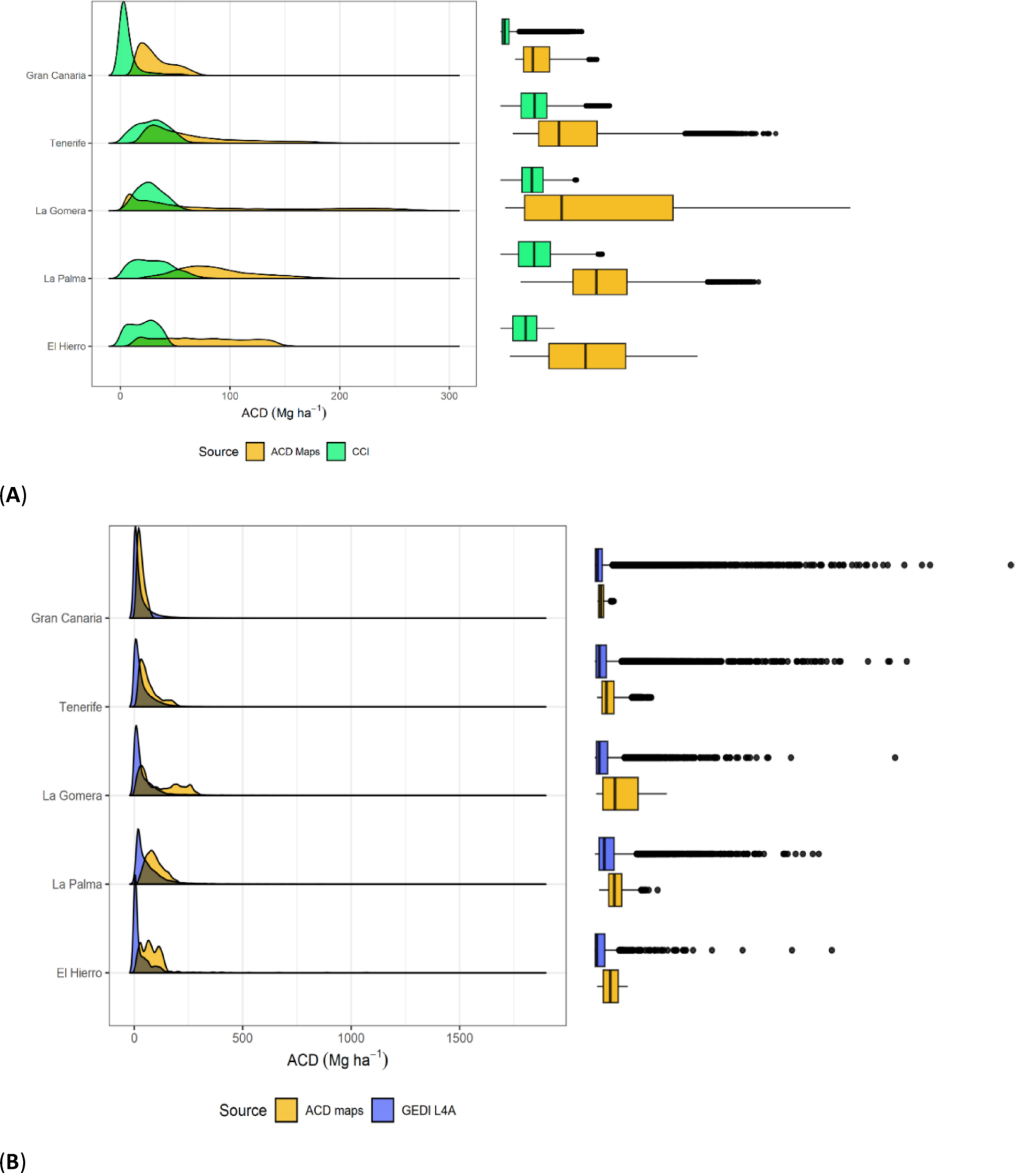
Comparison of frequency plots for above-ground carbon density (ACD) for each island and carbon estimation approach. **A**) Comparison of ACD maps generated in this study with ESA Climate Change Initiative CCI raster above ground biomass maps (scaled to ACD) based on 100x100m grid cells, **B**) Comparison of ACD maps generated in this study with laser-sampled ground locations from NASA’s GEDI L4A mission with estimates of above ground biomass (scaled to ACD, GEDI = Global Ecosystem Dynamics Investigation) and based on 25 m footprints. ACD distribution of grid cells or footprints shown by frequency distribution (left) and boxplots (right)

When comparing our carbon density maps with other biomass products, both the ESA CCI Above-Ground Biomass maps and the Global Ecosystem Dynamics Investigation (GEDI) Level 4 A (L4A) footprints yielded substantially lower mean carbon density values at the island level than those estimated in the present study (Fig. 7). The CCI product exhibited lower maximum carbon density values, whereas GEDI produced considerably higher maxima, including extremely high and unrealistic estimates exceeding 1500 Mg ha^− 1^, which were much higher than those obstained in this study. The best match between GEDI estimates and our results was observed for Gran Canaria except for high density values.

## Discussion

### Carbon stocks and densities

Our study aimed to develop a high-resolution carbon storage map for all forest types on the entire Canarian archipelago by integrating updated field inventory data, remote sensing variables and robust statistical models. The objective was to provide environmental researchers, policymakers, and forest management professionals with accurate information to improve conservation strategies, carbon sequestration efforts, and sustainable forest management practices. This method could be applied to other oceanic islands where forest inventory data are available.

Overall, our results revealed higher overall TCSs and TCDs than previous estimates for Canarian forests. An earlier estimate of total forest carbon stocks for the Canary Islands produced by MITECO [46], based only on tree data, using species-specific but different allometric equations and simple spatial extrapolation factors (mean forest carbon density × total forest area), reported a TCS of 8.23 Tg [46], compared with 10.26 Tg TCS estimated in this study. The aboveground carbon stock of 7.5 Tg ACS calculated for 112,735 ha forest cover on the Canary Islands corresponds to 66.5 Mg C ha^− 1^ ACD, which is comparable to the 64.0 Mg C ha^− 1^ ACD reported for Hawaii Island or 67.1 Mg C ha^− 1^ ACD for Maui Island, both values were obtained for tropical island forests [14].

Previous stand-level studies in the Canaries reported lower biomass and carbon densities. For example, Aboal et al. [44] found 210–240 Mg ha^− 1^ AGB (equivalent to approximately 105–120 Mg C ha^− 1^ ACD) for laurel forests on Tenerife and La Gomera, while MITECO [46] reported mean total carbon density (TCD) of 135 Mg C ha^− 1^ for the Canarian laurel forest. In contrast, our study estimated mean values ranging from 130 to 270 Mg C ha⁻¹ for ACD and from 180 to 410 Mg C ha⁻¹ for TCD in these subtropical evergreen forests. Similarly, our carbon density estimates for *Erica–Morella* woodlands, pine forests, and thermophilous woodlands were generally higher than those reported in previous studies [46, 48]. These discrepancies are likely reflect the more recent and accurate methods used here. The accuracy of a carbon stock model depends on factors such as the type of allometric equation, available forest plot data, selection and availability of predictors, and statistical tools [83], and biases related to each factor can accumulate. We therefore selected the most appropriate and robust estimation techniques available. Nevertheless, among the potential limitations of our approach is the lack of species-specific allometric equations for several laurel forest tree species, particularly for *Persea indica*, which is abundant in some variants of laurel forest, as well as the absence of certain abiotic and biotic predictors capable of capturing the high spatial heterogeneity of structural and ecological factors such as soil variables in these topographically complex islands. Incorporating such predictors would likely enhance predictive performance by improving both the accuracy of field biomass data used for calibration and the selection of ecologically relevant predictors for statistical models.

As a result, Canarian laurel forests reached a carbon density comparable to the highest ACD values reported for forests across the Hawaiian archipelago (300–500 Mg C ha^-1^ ACD [14]) and exceeded values documented for many subtropical and tropical regions [6, 26, 84, 85]. Notably, these estimates do not yet include carbon stored in deadwood, litter, or soil. Deadwood carbon stock for Canarian laurel forest has been estimated at about 2 Mg C ha^− 1^ [47], forest floor carbon in Spanish woodlands at 5–15 Mg C ha^-1^ [23], and soil carbon in Canarian laurel forest at 150–200 Mg C ha^-1^ [86]. Conserved, mature pine and laurel forest stands in this study can exceed 300 Mg C ha^− 1^ TCD, matching the highest values observed for European and temperate old-growth systems [29, 87]. Typical Iberian forests accumulate much less carbon [17, 88, 89], rising to about 200 Mg C ha^− 1^ TCD only in broadleaved hardwood forests in northern Spain [5, 90]. Furthermore, ACD values between 38.4 Mg C ha^− 1^ (SE Europe) and 93 Mg C ha^− 1^ (Western Europe) have been reported for other European forests [91]. Laurel forests on other Macaronesian islands exhibit lower aboveground carbon density (ACD) values than those found in the Canary Islands. Reported values include 67 Mg C ha⁻¹ ACD in Madeira and 266 Mg ha⁻¹ AGB (equivalent to approximately 130 Mg C ha⁻¹ ACD) in the Azores, compared with 130–270 Mg C ha⁻¹ ACD estimated for the Canary Islands [41, 42]. Potential explanations for these differences include ecological factors, such as climate and site productivity, methodological differences related to biomass estimation techniques, allometric equations, and plot selection, as well as varying degrees of habitat degradation. Further research is needed to clarify the drivers of variation in carbon stocks across Macaronesian laurel forests. Our findings underscore the importance of the Canary Islands for carbon sequestration in laurel forests, a habitat type of Community interest under the EU Habitats Directive.

### Predictor variables

We found that TCSs vary greatly among islands and forest types, and even within the same forest type, a pattern that can be linked to structural, dynamic, and ecological factors. Among the predictors considered, the most powerful variables for estimating TCDs on the Canary Islands were stand structural attributes derived from LiDAR (tree canopy cover, volume, and height), forest type and a satellite-based moisture index reflecting vegetation water content, consistent with studies from other regions [14, 23, 92]. LiDAR-derived metrics have been widely and successfully used to estimate AGB and TCSs [13, 15–17, 66, 92]. Previous studies have shown that forest structural attributes reflect not only site productivity but also successional dynamics and management history, all of which influence biomass accumulation and carbon storage [93]. Forest age is a key determinant of carbon storage [94]: in early to mid-successional stages, biomass and carbon stocks generally increase with forest age, depending on disturbance regime and resource availability [27, 87, 95]. Forest management practices such as thinning can further affect carbon sequestration in Mediterranean forests [11].

Water availability is another major driver of productivity, biomass, and carbon accumulation, especially in lowlands or mid-elevations where temperature is not a strongly limiting factor. Consequently, humid forests generally store more carbon than mesic or dry forests and savanna woodlands [87, 96, 97]. Precipitation has repeatedly been identified as a fundamental control on aboveground forest biomass and carbon accumulation [98, 99]. Soil moisture also controls the partitioning of TCSs, showing a positive relationship with soil carbon pools but a unimodal relationship with ACSs in boreal forests [100]. In our analysis, precipitation became a more important predictor of TCD when structural data were excluded, reflecting co-variation among variables, for example, summer rainfall on Gran Canaria, or annual precipitation on La Gomera (Table S8). In addition to the moisture index, Sentinel 2 A band b12 or SWIR2, which captures short-wave infrared reflectance and is sensitive to vegetation and soil water stress, was also an excellent predictor for TCSs on four of the five Canary Islands. Notably, both were satellite-derived variables and yielded higher importance values than the climatic variables interpolated from ground-based meteorological stations. Moisture or precipitation was therefore strongly related to forest TCD in the Canaries, whereas temperature, despite spanning a broad range (9–22 °C), was not. Mean annual temperature showed only a weak unimodal relationship with TCS peaking at mid-elevations that coincide with the potential laurel forest belt, a pattern similar to that observed in Hawaii across a narrower temperature range of 5.2 °C [101].

Considering moisture availability and structural dynamics, the current distribution of forest carbon pools in the Canary Islands can be interpreted as follows. The lowest TCDs occur in the most disturbed or youngest stands at the driest sites, such as low-elevation thermophilous woodlands recovering from intensive agriculture or timber extraction [50, 53]. The small overall contribution of thermophilous woodlands to the archipelago’s TCS (1.1%, 0–4.6% depending on island) reflects their historical destruction and degradation, rather than an intrinsically low storage capacity (20–70 Mg C ha⁻¹ TCD for open, small-stemmed stands [50, 53]). In contrast, the most humid and least disturbed sites, with the highest TCDs, are dominated by mature laurel forest or humid pine forest located at the mid-elevations of the windward slopes of the islands. Natural pine forests on Gran Canaria showed the lowest TCD (mean TCD, 22–52 Mg C ha⁻¹) probably due to both generally drier conditions and higher levels of historical exploitation and degradation, whereas natural pine forests on La Palma, the wettest island with the best conserved stands, reached the highest TCDs (73–150 Mg C ha⁻¹ between dry and humid pine forest), roughly threefold higher. A similar pattern appears in laurel forest: dry, and secondary laurel forest stands on Tenerife, still recovering from past exploitation and dominated by young trees [52], showed the lowest TCDs (mean TCD, 56 Mg C ha⁻¹), while old-growth, humid laurel forest in Garajonay National Park (La Gomera) reaches exceptionally high TCDs (mean TCD, 413 Mg C ha⁻¹). High-resolution carbon mapping thus reveals how disturbance regimes interact with climate to shape carbon accumulation, even though detailed stand-age reconstructions for each island are still lacking.

The contribution of exotic tree plantations to TCSs in the Canarian archipelago is relatively low (5%), except for Tenerife and Gran Canaria, where these stands account for higher proportions (8–17%). However, *Castanea sativa*, *Eucalyptus,* and *Pinus radiata* plantations can locally store up to 200 Mg C ha^− 1^ depending on stand density and age, similar to values reported for these formations in north-western Spain [16]. *Pinus radiata* and *Eucalyptus globulus* have not been reported as invasive in the Canary Islands, as they do not spread beyond reforested sites, although both have been extensively planted, and *Eucalyptus* can be difficult to eradicate. In contrast, *Castanea sativa* appears to have a certain potential to invade disturbed laurel forest on La Palma, where it may form a novel ecosystem [102].

Overall, invasive tree species in the Canaries are not considered a major threat to native forests [103] and do not generally reduce native forest TCSs, unlike on other oceanic islands such as Hawaii, where alien trees can substantially modify native forest structure [104] and carbon storage, depending on ecological conditions, species traits, and geological age [105]. In fact, *Morella faya*, a pioneer tree forming the *Erica-Morella* forest on the Canaries, extensively invades young lava flows on Hawaii owing to its N-fixing capacity [106].

In the context of climate change, our findings suggest that the size and distribution of forest carbon pools on the Canary Islands are likely to be less sensitive to rising mean annual temperature under global warming than other climate change consequences, such as water availability. Potential carbon losses at lower altitudes might be offset by gains at higher altitudes. Indeed, pine forests are currently expanding in area and shifting the tree line upwards at high elevations on Tenerife [107]. Climate change is projected to increase the duration and intensity of droughts across the Canary Islands [108], especially at higher elevations, which may have negative consequences for the islands’ forest carbon stocks.

However, changes in Canarian forest TCSs will probably be driven more by successional processes linked to human land-use change and forest management than by climate change per se. Since the mid-20th century, pine and especially laurel forests have regenerated spontaneously on abandoned land (passive restoration) at mid-elevations on the larger islands, following the abandonment of agriculture at these elevations and a shift toward tourism-based economic activities concentrated along the coast [52]. Important gains in TCSs can also be expected from recent and future ecological restoration projects that aim to convert exotic tree plantations, such as those of *Pinus radiata* and *Eucalyptus globulus*, into natural laurel forests within their potential distribution areas [109, 110].

### Model comparison

Both modelling approaches, BRT and RF, applied here performed similarly in terms of efficiency, accuracy and error statistics, and the best fitted models depended on the island. Although we detected six important predictors common to all islands, the ranking of remaining explanatory variables differed strongly between islands. This indicates that carbon mapping should be conducted separately for each island rather than using a single archipelago-wide model, which would entail a loss of accuracy. The predictive power of the models was high for Tenerife, La Gomera and El Hierro, with high coefficients of determination R^2^ > 0.65, comparable to validation statistics reported in similar studies [13, 14, 20]. In contrast, the models for La Palma and Gran Canaria showed moderate predictive performance (R^2^ = 0.35–0.42), suggesting that some key ecological factors or structural parameters were not captured. On La Palma, high TCDs occur across several native forest types (*Erica-Morella* forests, laurel and pine forests), which were largely independent of moisture and canopy cover. More detailed stand age analyses might clarify the role of additional structural attributes, such as the vertical stratification and spatial distribution of large trees, in determining TCDs. Big-sized trees have been identified as a crucial driver of forest functioning and biomass accumulation [111]. LiDAR data have proved very useful in estimating forest biomass and TCD, but their acquisition is costly compared with freely available satellite data such as Landsat and Sentinel [14, 15, 92]. In our study, models without LiDAR data still achieved acceptable performances (Table S9). For example, the RF model for TCD on La Gomera showed only a small decrease in predictive ability (R^2^ = 0.78 to R^2^ = 0.74), suggesting that, in the absence of LiDAR data, carbon mapping remains feasible when robust cross-validation with field data is available.

The NFI plot data used here are extremely valuable since they come from a systematic, grid-based forest plot inventory. This design is highly advantageous for aboveground carbon mapping, as it provides a high number of spatially unbiased training and validation datasets and helps machine-learning models to capture large-scale gradients in biomass and carbon accumulation [112]. Therefore, forest monitoring plots have frequently been used to produce large-scale estimates of TCSs in Europe [83] and other regions [113]. However, a drawback of this approach is that rare habitats are underrepresented, whereas very common ones are overrepresented. This may explain why NFI plots assigned to a specific forest type may be absent on a given island, even though that formation still occurs there in small, fragmented patches (e.g. thermophilous woodlands on La Palma), but it was not intersected by the systematic sampling grid.

### Comparison with other biomass products

Overall, both the CCI and GEDI biomass products substantially underestimated forest carbon density relative to the estimates derived in this study and are therefore not suitable for estimating carbon stocks across the Canary Islands. However, in specific cases, such as Gran Canaria, GEDI-derived estimates may provide useful approximations if unrealistically high values are excluded from the analysis.

The ESA Climate Change Initiative (CCI) and GEDI 4LA datasets use complementary approaches to estimate AGB, both relying on ground-based validation. The CCI Biomass maps combine spaceborne Synthetic Aperture Radar (SAR) and optical data within a machine-learning framework calibrated using National Forest Inventories (NFIs) and scientific plot networks. In contrast, GEDI-4LA uses spaceborne LiDAR-derived canopy height metrics to generate high-resolution biomass estimates, with models trained and validated using local forest plots to capture complex forest structure. However, calibrating high-resolution GEDI L4A footprints with NFI plots remains challenging [114].

In general, several critical factors must be considered when using ALS-derived structural data or global spaceborne metrics as predictors for estimating above-ground biomass density. Biomass calibration with field data can be influenced by various sources of uncertainty, including the spatial scale of ALS measurements, geolocation discrepancies between response and predictor variables, temporal mismatches and seasonal effects, as well as differences in the spatial resolution of remote sensing data [115].

### Opportunities for forest management and land-use planning

High-resolution carbon mapping can be used to evaluate current protections, threats and opportunities for carbon sequestration and to guide conservation-based forest management. The carbon stock maps generated in this study might be evaluated and related to forest protection status, past and present land use change and biodiversity conservation. For instance, the overlapping areas of the current spatial distribution of high TCDs and the Canary Islands Network for Protected Natural Areas could inform where protection should be expanded. In this context, old-growth forests should be identified and urgently protected, given their exceptional carbon storage capacity.

Furthermore, island-level analyses about carbon stocks and land use changes could also highlight opportunities to increase carbon storage through ecological succession, such as spontaneous forest recovery on abandoned land within potential forest areas, or through reforestation programs. To enhance carbon stocks, passive restoration should be promoted where it is already occurring, and reforestation prioritized in more humid sites within each forest type’s potential range to reduce climate-change risks [52, 53].

In line with nature-based forest management, dense pine plantations should be gradually converted into more natural stands and, ultimately, old-growth forests to improve carbon stocks and ecosystem functioning, recognizing that TCD may initially decline after thinning before increasing again [11]. Overall, the relationships among forest structure, carbon fluxes and biodiversity in the Canary Islands should be investigated in detail to better inform forest and land management, including other carbon pools such as deadwood, the forest floor and soil carbon.

## Conclusions

We have shown that our approach, which combines NFI field data with advanced remote sensing tools, including LiDAR and Sentinel-2 multispectral imagery and machine learning algorithms, was successful in quantifying both AGB and BGB for all major forest types and produced a high-resolution carbon map. It therefore provides an effective methodological framework for carbon assessment on topographically complex oceanic islands. Both BRT and RF models performed similarly in estimating carbon stocks in terms of goodness-of-fit statistics.

Our study revealed that the Canary Islands harbor extraordinary TCDs, particularly within the mature humid laurel forests of La Gomera and identified stand structural characteristics such as tree canopy cover and tree volume derived from LiDAR as the primary drivers of spatial variation in carbon stocks. In addition, water availability and moisture indices emerged as stronger controls of carbon accumulation than temperature, suggesting that these carbon pools may be relatively resilient to increasing mean annual temperatures in the context of climate change, while remaining sensitive to shifts in precipitation regimes and land-use dynamics.

Finally, these high-resolution maps offer an important baseline for sustainable forest management, biodiversity conservation, and regional climate policy. Our findings underscore the importance of protecting mature, old growth stands and can inform strategies to optimize carbon sequestration through targeted reforestation and passive restoration, thereby advancing carbon neutrality goals.

## Supplementary Information

Supplementary Material 1

Supplementary Material 2

## Data Availability

The data that supports the findings of this study are available in the supplementary material of this article. Raw data of the National Forest Inventory of Spain is available at: https://www.miteco.gob.es/es/biodiversidad/temas/inventarios-nacionales/inventario-forestal-nacional/cuarto_inventario.html.

## Abbreviations

ACD: Aboveground carbon density
ACS: Aboveground carbon stock
AGB: Aboveground biomass
BCD: Belowground carbon density
BCS: Belowground carbon stock
BGB: Belowground biomass
BRT: Boosted regression tree
C: Gran Canaria
CD: Carbon density
dbh: Diameter at breast height
F: Fuerteventura
G: La Gomera
GAM: Generalized additive models
H: El Hierro
L: Lanzarote
LiDAR: Light detection and ranging
MAE: Mean absolute error
MITECO: Spanish Ministry for Ecological Transition and the Demographic Challenge
NDMI: Normalized difference moisture index
NDVI: Normalized difference vegetation index
NDWI: Normalized difference water index
NIF: Spanish National Forest Inventory
P: La Palma
R²: Coefficient of determination
RF: Random forest
RMSE: Root mean squared error
SWIR: Short wave infrared
T: Tenerife
TCD: Total carbon density
TCS: Total carbon stock
