# Supplementary material for: Modelling forest carbon stocks on the Canary Islands
Source: Carbon Balance Manag. 2026 Jul 27;21:104. doi: 10.1186/s13021-026-00488-4 (PMC13452119; doi:10.1186/s13021-026-00488-4)
Supplement: Supplementary file 1 [file 13021_2026_488_MOESM1_ESM.docx]

Table S1. Precipitation charactersitics (mm) (minimum, maximum, mean, median value) for laurel and pine forest types in the Canary Islands.

| El Hierro | Min | Max | Mean | Median |
| --- | --- | --- | --- | --- |
| Typical pine forest | 268 | 692 | 571 | 588 |
| Dry laurel forest | 30 | 713 | 483 | 475 |
| Typical laurel forest | 469 | 564 | 510 | 508 |
| La Palma | Min | Max | Mean | Median |
| Typical pine forest | 317 | 1510 | 795 | 769 |
| Dry pine forest | 257 | 919 | 530 | 536 |
| Humid pine forest | 454 | 1510 | 939 | 991 |
| High mountain pine forest | 547 | 1374 | 979 | 991 |
| Dry laurel forest | 366 | 1140 | 705 | 705 |
| Typical laurel forest | 473 | 1474 | 959 | 945 |
| Humid laurel forest | 629 | 1329 | 874 | 846 |
| La Gomera | Min | Max | Mean | Median |
| Typical pine forest | 385 | 526 | 434 | 425 |
| Dry laurel forest | 184 | 791 | 478 | 480 |
| Typical laurel forest | 297 | 829 | 657 | 672 |
| Humid laurel forest | 448 | 829 | 664 | 664 |
| Tenerife | Min | Max | Mean | Median |
| Typical pine forest | 200 | 866 | 370 | 354 |
| Dry pine forest | 170 | 940 | 365 | 317 |
| Humid pine forest | 249 | 950 | 562 | 545 |
| High mountain pine forest | 248 | 585 | 404 | 411 |
| Dry laurel forest | 209 | 728 | 399 | 374 |
| Typical laurel forest | 310 | 764 | 529 | 524 |
| Gran Canaria | Min | Max | Mean | Median |
| Typical pine forest | 194 | 685 | 369 | 369 |
| Humid pine forest | 290 | 590 | 480 | 474 |
| Dry laurel forest | 180 | 680 | 411 | 402 |
| Typical laurel forest | 440 | 478 | 467 | 465 |
| Humid laurel forest | 440 | 723 | 566 | 566 |

Table S2. Allometric equations used for calculating the aboveground and belowground biomass and carbon density of the tree stratum.

| Species | Allometric equation asigned | Source |
| --- | --- | --- |
| *Acacia dealbata* | *Acacia dealbata* | MITECO 2021 |
| *Acacia spp.* | *Acacia spp.* | MITECO 2021 |
| *Apollonias barbujana* | *Other laurel forest species* | Menéndez-Miguélez et al. 2021 |
| *Arbutus canariensis* | *Other laurel forest species* | Menéndez-Miguélez et al. 2021 |
| *Castanea sativa* | *Castanea sativa* | Menéndez-Miguélez et al. 2021 |
| *Cedrus atlantica* | *Cedrus atlantica* | MITECO 2021 |
| *Ceratonia siliqua* | *Ceratonia siliqua* | MITECO 2021 |
| *Cupressus macrocarpa* | *Cupressus macrocarpa* | MITECO 2021 |
| *Cupressus sempervirens* | *Cupressus sempervirens* | MITECO 2021 |
| *Erica arborea* | *Erica arborea* | Menéndez-Miguélez et al. 2021 |
| *Erica scoparia* | *Erica arborea* | Menéndez-Miguélez et al. 2021 |
| *Eucalyptus camaldulensis* | *Eucalyptus camaldulensis* | MITECO 2021 |
| *Eucalyptus globulus* | *Eucalyptus globulus* | MITECO 2021 |
| *Eucalyptus nitens* | *Eucalyptus nitens* | MITECO 2021 |
| *Ficus carica* | *Ficus carica* | MITECO 2021 |
| *Heberdenia bahamensis* | *Other laurel forest species* | Menéndez-Miguélez et al. 2021 |
| *Ilex canariensis* | *Ilex canariensis* | Menéndez-Miguélez et al. 2021 |
| *Ilex platyphylla* | *Ilex canariensis* | Menéndez-Miguélez et al. 2021 |
| *Juglans regia* | *Juglans regia* | MITECO 2021 |
| *Juniperus cedrus* | *Juniperus oxycedrus* | Menéndez-Miguélez et al. 2021 |
| *Juniperus turbinata* | *Juniperus phoenicea* | Menéndez-Miguélez et al. 2021 |
| *Laurus azorica/novocanariensis* | *Laurus azorica/novocanariensis* | Menéndez-Miguélez et al. 2021 |
| *Laurus nobilis* | *Laurus nobilis* | Menéndez-Miguélez et al. 2021 |
| *Malus sylvestris* | *Malus sylvestris* | MITECO 2021 |
| *Myrica faya* | *Myrica faya* | Menéndez-Miguélez et al. 2021 |
| *Ocotea phoetens* | *Other laurel forest species* | Menéndez-Miguélez et al. 2021 |
| *Olea europaea* | *Olea europaea* | Menéndez-Miguélez et al. 2021 |
| *Persea indica* | *Other laurel forest species* | Menéndez-Miguélez et al. 2021 |
| *Phoenix canariensis* | *Phoenix dactylifera* | Issa et al. 2018 |
| *Phoenix spp.* | *Phoenix dactylifera* | Issa et al. 2018 |
| *Picconia excelsa* | *Other laurel forest species* | Menéndez-Miguélez et al. 2021 |
| *Pinus canariensis* | *Pinus canariensis* | Menéndez-Miguélez et al. 2021 |
| *Pinus halepensis* | *Pinus halepensis* | MITECO 2021 |
| *Pinus pinaster* | *Pinus pinaster* | Menéndez-Miguélez et al. 2021 |
| *Pinus pinea* | *Pinus pinea* | Menéndez-Miguélez et al. 2021 |
| *Pinus radiata* | *Pinus radiata* | Menéndez-Miguélez et al. 2021 |
| *Populus alba* | *Populus alba* | MITECO 2021 |
| *Populus nigra* | *Populus nigra* | MITECO 2021 |
| *Prunus avium* | *Prunus avium* | MITECO 2021 |
| *Prunus lusitanica* | *Other laurel forest species* | Menéndez-Miguélez et al. 2021 |
| *Prunus spp.* | *Other laurel forest species* | Menéndez-Miguélez et al. 2021 |
| *Pyrus spp.* | *Malus sylvestris* | MITECO 2021 |
| *Quercus robur* | *Quercus robur* | Menéndez-Miguélez et al. 2021 |
| *Rhamnus glandulosa* | *Other laurel forest species* | Menéndez-Miguélez et al. 2021 |
| *Robinia pseudoacacia* | *Robinia pseudoacacia* | MITECO 2021 |
| *Salix canariensis* | *Other laurel forest species* | Menéndez-Miguélez et al. 2021 |
| *Salix spp.* | *Salix spp.* | MITECO 2021 |
| *Tamarix canariensis* | *Tamarix canariensis* | MITECO 2021 |
| *Ulmus minor* | *Ulmus minor* | MITECO 2021 |
| *Visnea mocanera* | *Other laurel forest species* | Menéndez-Miguélez et al. 2021 |

Table S3. Allometric equations used for calculating the aboveground and belowground biomass and carbon density of the shrub stratum.

| Species cited in NFI | Species of the Canary flora asigned | Allometric equation asigned | Source |
| --- | --- | --- | --- |
| *Adenocarpus spp.* | *Adenocarpus foliolosus* | *Adenocarpus foliosus* | BEI group |
|  | *Adenocarpus viscosus* |  |  |
| *Artemisia spp.* | *Artemisia thuscula* | *Artemisia thuscula* | BEI group |
| *Asparagus spp.* | *Asparagus umbellatus* | Setos, bardas, orlas | Montero et al. 2020 |
|  | *Asparagus scoparius* |  |  |
| *Cistus ladanifer* | *Cistus symphytifolius* | *Cistus albus* | Montero et al. 2020 |
| *Cistus monspeliensis* | *Cistus monspeliensis* | *Cistus monspeliensis* | Elaboración propia |
| *Cistus scoparius* | *Cistus scoparius* | *Cistus scoparius* | Montero et al. 2020 |
| *Cistus spp.* | *Cistus creticus* | *Cistus creticus* | Montero et al. 2020 |
| *Daphne gnidum* | *Daphne gnidum* | *Erica arborea* | Montero et al. 2020 |
| *Dophnea spp.* | *Daphne gnidium* | *Erica arborea* | Montero et al. 2006 |
| *Erica arborea* | *Erica arborea* | *Erica arborea* | Montero et al. 2006 |
| *Lavandula spp.* | *Lavandula canariensis* | *Lavandula stoechas* | Montero et al. 2006 |
| *Lavandula canariense* | *Lavandula canariensis* | *Lavandula stoechas* | Montero et al. 2006 |
| *Phillyrea angustifolia* | *Phillyrea angustifolia* | Mancha matorral y afines | Montero et al. 2006 |
| *Retama monosperma* | *Retama monosperma*  *Retama odoratissima* | *Retama sphaerocarpa* | Montero et al. 2020 |
| *Retama rhodorhizoides* | *Retama rhodorhizoides* | *Retama sphaerocarpa* | Montero et al. 2020 |
| *Rhamus spp.* | *Rhamus crenulata*  *Rhamnus integrifollia* | Setos, bardas, orlas | Montero et al. 2020 |
| *Rosa spp.* | *Rosa spp.* | Setos, bardas, orlas | Montero et al. 2020 |
| *Spartium junceum* | *Spartium junceum* | Matorrales leguminosas retamoideas | Montero et al. 2020 |
| *Teline spp.* | *Teline spp.* | Matorrales leguminosas retamoideas | Montero et al. 2020 |
| *Thymus spp.* | *Micromeria spp.* | *Thymus spp.* | Montero et al. 2020 |
| *Ulex europaeus* | *Ulex europaeus* | *Ulex europaeus* | Montero et al. 2020 |
| *Ulex spp.* | *Ulex europaeus* | *Ulex europaeus* | Montero et al. 2020 |
| *Viburnum spp.* | *Viburnum rigidum* | *Erica arborea* | Montero et al. 2020 |
| *Viburnum tinus* | *Viburnum rugosum* | *Erica arborea* | Montero et al. 2020 |

Tabla S4. Ratio between belowground biomass (BGB) / aboveground biomass (AGB) (root/shoot ratio) for the tree species occurring in the Canary Island forest ecosystems (Montero et al. 2005, Issa et al. 2019). For species where no value is available, we applied data from a congeneric species on the Iberian Peninsula, such as *Juniperus turbinata*, for which we used the value from *Juniperus phoenicea*. Similarly, for *Juniperus cedrus*, we applied the value from *Juniperus oxycedrus*. For species from the laurel forest lacking data, an average value from the studied laurel forest species was applied. In the case of broadleaf species and other conifers, their respective averages were also used.

| Species | Species in references | BGB/AGB |
| --- | --- | --- |
| *Castanea sativa* | *Castanea sativa* | 0.87 |
| *Erica arborea* | *Erica arborea* | 0.44 |
| *Eucalyptus spp.* | *Eucalyptus spp.* | 0.49 |
| *Ilex canariensis* | *Ilex canariensis* | 0.65 |
| *Juniperus cedrus* | *Juniperus oxycedrus* | 0.59 |
| *Juniperus turbinata* | *Juniperus phoenicea* | 0.55 |
| *Laurus novocanariensis* | *Laurus novocanariensis* | 0.4 |
| *Myrica (Morella) faya* | *Myrica faya* | 0.64 |
| *Olea europaea* | *Olea europaea* | 0.46 |
| *Pinus canariensis* | *Pinus canariensis* | 0.26 |
| *Pinus halepensis* | *Pinus halepensis* | 0.31 |
| *Pinus radiata* | *Pinus radiata* | 0.27 |
| *Cupressus macrocarpa* | Other conifers | 0.39 |
| *Robinia pseudoacacia* |  |  |
| *Tamarix canariensis* |  |  |
| *Ficus carica* | Other broad leaved species | 0.54 |
| *Prunus avium* |  |  |
| *Apollonias barbujana* |  |  |
| *Arbutus canariensis* |  |  |
| *Pistacia atlantica* |  |  |
| *Prunus spp.* | Other laurel forest species | 0.48 |
| *Prunus lusitanica* |  |  |
| *Visnea mocanera* |  |  |
| *Salix canariensis* |  |  |
| Other laurel forest species |  |  |

Table S5. Belowground biomass/aboveground biomass ratio (BGB/AGB) (root/shoot ratio) of the species present in the understory of Canarian forest ecosystems recorded at the permanent plots of the National Forest Inventory of Spain (NFI).

| NFI Inventory Species | Canarian Species | % Carbon | BGB/AGB Ratio | Source |
| --- | --- | --- | --- | --- |
| *Adenocarpus spp.* | *Adenocarpus foliosus* | 52.4 | 0.427 | Vega 2023 |
|  | *Adenocarpus viscosus* |  |  |  |
| *Artemisia spp.* | *Artemisia thuscula* |  | 0.515 | Vega 2023 |
| *Asparagus spp.* | *Asparagus umbellatus* | 49.4 | 0.427 | Vega 2023 |
|  | *Asparagus scoparius* |  |  |  |
| *Cistus ladanifer* | *Cistus symphytifolius* | 49.5 | 0.48 | Vega 2023 |
| *Cistus monspeliensis* | *Cistus monspeliensis* | 49.6 | 0.48 | Vega 2023 |
| *Cytisus scoparius* | *Cytisus scoparius* | 50.2 | 0.4 | Vega 2023 |
| *Cytisus spp.* | *Chamaecytisus proliferus* | 50.2 | 0.4 | Burrows et al. 2015 |
| *Daphne gnidium* | *Daphne gnidium* | 51.2 | 0.479 | Montero et al. 2005 |
| *Daphne spp.* | *Daphne gnidium* | 51.2 | 0.479 | Montero et al. 2005 |
| *Erica arborea* | *Erica arborea* | 51.4 | 0.443 | Montero et al. 2005 |
| *Lavandula spp.* | *Lavandula canariensis* | 53.1 | 0.61 | Vega 2023 |
| *Lavandula stoechas* | *Lavandula canariensis* | 53.1 | 0.61 | Vega 2023 |
| *Phillyrea angustifolia* | *Phillyrea angustifolia* | 53 | 0.7 | Vega 2023 |
| *Pistacia lentiscus* | *Pistacia lentiscus* | 49.3 | 0.9 | Vega 2023 |
| *Retama rhodorhizoides* | *Retama rhodorhizoides* | 50.4 | 1.5 | Ruiz-Peinado et al. 2013 |
| *Rhamnus spp.* | *Rhamnus crenulata* | 48.6 | 0.8 | Vega 2023 |
|  | *Rhamnus integrifolia* |  |  |  |
| *Rosa spp.* | *Rosa spp.* | 49.7 | 0.427 | Vega 2023 |
| *Rubus spp.* | *Rubus ulmifolius* | 49.2 | 0.427 | Vega 2023 |

Table S6. Total forest cover and forest carbon stocks (aboveground and belowground) for each forest type and island. S: Juniper woodlands, P: Palm forest, FB: *Erica-Morella* forests, L: Typical laurel forest, LS: Dry laurel forest, LH: Humid laurel forest, PS: Dry Canarian pine forest, PT: Typical Canarian pine forest, PH: Humid Canarian pine forest, PAM: High-elevation Canarian pine forest, RDB: Low-density plantations of Canarian pine, RDM: Medium-density plantations of Canarian pine, RDA: High-density plantations of Canarian pine, PCF: Plantations of exotic conifers and broad-leaved species, PPR: Plantations of *Pinus radiata,* EUC: Plantations of *Eucalyptus* species, C: Plantations of chestnut (*Castanea sativa*), ACL: Wild Canarian olive tree woodlands.

| Forest type | Tenerife |  |  | Gran Canaria |  |  | La Gomera |  |  | La Palma |  |  | El Hierro |  |  | Canarias |  |  |
| --- | --- | --- | --- | --- | --- | --- | --- | --- | --- | --- | --- | --- | --- | --- | --- | --- | --- | --- |
|  | ha | BRT | RF | ha | BRT | RF | ha | BRT | RF | ha | BRT | RF | ha | BRT | RF | ha | BRT | RF |
| S | 143.5 | 4217.4 | 3367.7 |  | 0.0 | 0.0 | 2068.3 | 53191.4 | 44843.5 | 49.8 | 158.9 | 3059.0 | 822.5 | 28763.8 | 25216.6 | 3084 | 86331.5 | 76486.8 |
| ACL |  | 0 | 0 | 357.5 | 14002.1 | 13148.9 |  | 0 | 0 |  | 0 |  |  | 0 | 0 | 357.5 | 14002.1 | 13148.9 |
| P | 12.3 | 643.6 | 640.8 | 149.5 | 4644.6 | 4307.5 | 514.8 | 16190.3 | 11293.8 | 19.8 | 40.3 | 1163.2 |  | 0 | 0 | 696.3 | 21518.8 | 17405.2 |
| FB | 6870.3 | 593131.8 | 682021.4 | 56 | 3616.1 | 3540.5 | 3075.8 | 315185.2 | 294888.9 | 7618.5 | 722134.8 | 909556.2 | 1824 | 239203.3 | 253226.8 | 19444.5 | 1873271.1 | 2143233.9 |
| LH |  | 0 | 0 | 3 | 215.9 | 0 | 662 | 201259.5 | 233879.9 | 92.5 | 13974.8 | 13728.7 |  | 0 | 0 | 757.5 | 215450.2 | 247608.6 |
| L | 1167.8 | 183703.4 | 176147.4 | 103 | 6304.5 | 0 | 2214.3 | 576598.0 | 586360.4 | 3066 | 524868.5 | 518397.7 | 2.5 | 516.3 | 0 | 6553.5 | 1291990.7 | 1280905.5 |
| LS | 582.5 | 36996.9 | 40895.3 | 45.5 | 1632.2 | 1746.6 | 555.3 | 54223.3 | 57596.3 | 322.3 | 16901.7 | 34175.4 | 489.3 | 64742.0 | 70612.6 | 1994.8 | 174496.1 | 205026.0 |
| PH | 6460.3 | 708710.3 | 753682.5 | 658 | 35294.1 | 39580.3 |  | 0 | 0 | 3248.5 | 460532.4 | 475000.7 |  | 0 | 0 | 10366.8 | 1204536.8 | 1268263.5 |
| PT | 9819.3 | 473559.3 | 481729.6 | 8014 | 251269.9 | 220164.3 | 42 | 1576.1 | 1423.9 | 13695 | 1154246.4 | 1453381.2 | 1448.8 | 123159.8 | 126735.6 | 33019 | 2003811.6 | 2283434.6 |
| PS | 5888 | 259410.1 | 258097.0 |  | 0 | 0 |  | 106.3 | 0 | 4152.8 | 188281.1 | 316293.7 |  | 0 | 0 | 10040.8 | 447797.5 | 574390.7 |
| PAM | 3163.5 | 148627.0 | 136473.6 |  | 0 | 0 |  | 0 | 0 | 3401 | 203269.8 | 342419.2 |  | 0 | 0 | 6564.5 | 351896.8 | 478892.9 |
| RDA | 7074.8 | 854991.7 | 884855.6 | 962.3 | 58699.5 | 63379.4 |  | 2343.9 | 0 |  | 0 |  | 538.3 | 44672.9 | 45547.9 | 8575.3 | 960708.0 | 993782.9 |
| RDM | 2281.3 | 121360.4 | 112568.0 | 1051.3 | 50349.1 | 51765.1 | 505.5 | 35368.4 | 35579.1 |  | 0 |  | 5.5 | 266.8 | 0 | 3843.5 | 207344.7 | 199912.2 |
| RDB | 1304.3 | 77178.9 | 78248.0 | 280.5 | 13551.7 | 16386.9 | 48.5 | 1112.2 | 0 |  | 0 |  |  | 0 | 0 | 1633.3 | 91842.9 | 94634.9 |
| EUC | 464 | 45264.7 | 48196.3 | 510.8 | 28241.1 | 31725.2 |  | 0 | 0 | 0.5 | 0 | 23.2 | 0.8 | 34.6 | 0 | 976 | 73540.4 | 79944.6 |
| PPR | 1606.8 | 189499.2 | 171287.5 | 153.5 | 8941.2 | 9563.8 | 37 | 1980.7 | 1360.4 |  | 0 |  | 193 | 17967.3 | 20087.5 | 1990.3 | 218388.4 | 202299.1 |
| PCF | 438 | 42287.1 | 40737.2 | 758.3 | 40250.5 | 45608.3 | 291 | 9289.1 | 8616.2 | 173.5 | 23182.9 | 25074.8 | 165.8 | 10150.5 | 8979.9 | 1826.5 | 125160.2 | 129016.5 |
| C | 349 | 27717.1 | 27497.1 | 225 | 11226.0 | 14760.4 |  | 0 | 0 | 436.8 | 38851.2 | 47318.4 |  | 0 | 0 | 1010.8 | 77794.3 | 89576.0 |
| Total | 47625.7 | 3767298.8 | 3896444.9 | 13328.2 | 528238.6 | 515677.2 | 10014.5 | 1268424.7 | 1275842.4 | 36277.0 | 3346442.8 | 4139591.4 | 5490.5 | 529477.3 | 550406.7 | 112734.9 | 9439882.2 | 10377962.5 |
| % total | 42.2 | 39.9 | 37.5 | 11.8 | 5.6 | 5.0 | 8.9 | 13.4 | 12.3 | 32.2 | 35.5 | 39.9 | 4.9 | 5.6 | 5.3 | 100.0 | 100.0 | 100.0 |

Table S7. Mean values and standard deviations of aboveground (ACD), belowground (BCD) and total (TCD) carbon densities (Mg ha^-1^) for each forest type and island. N = number of IFN plots used. Abbreviations follow Table S5.

| Forest type | | Tenerife |  |  |  | Gran Canaria |  |  |  | La Gomera |  |  |
| --- | --- | --- | --- | --- | --- | --- | --- | --- | --- | --- | --- | --- |
|  | N | ACD | BCD | TCD | N | ACD | BCD | TCD | N | ACD | BCD | TCD |
| S | 4 | 8.0 ± 5.5 | 4 ± 2.2 | 12 ± 7.7 | 0 | 0 | 0 | 0 | 37 | 5.4 ± 3.8 | 4.8 ± 4.1 | 10.2 ± 7.5 |
| ACL | 0 | 0 | 0 | 0 | 24 | 20.7 ± 10.8 | 9.5 ± 6.4 | 30.2 ± 15.1 | 0 | 0 | 0 | 0 |
| P | 2 | 47.6 ± 9.1 | 21.6 ± 2.9 | 69.2 ± 6.2 | 3 | 24.1 ± 7.61 | 10.6 ± 6.4 | 34.7 ± 9.5 | 19 | 12.4 ± 17.9 | 7.1 ± 10 | 19.4 ± 27.8 |
| FB | 78 | 65.1 ± 50 | 39.7 ± 26.5 | 93.9 ± 72.4 | 3 | 34.8 ± 16 | 9.5 ± 4.7 | 44.3 ± 20.4 | 52 | 49.9 ± 50.3 | 37.4 ± 29.7 | 87.4 ± 78.1 |
| LH | 0 | 0 | 0 | 0 | 0 | 0 | 0 | 0 | 12 | 270.4 ± 101.1 | 142.8 ± 49.1 | 413.2 ± 149.5 |
| L | 27 | 129.8 ± 73.4 | 62.4 ± 35.2 | 180.6 ± 107.7 | 1 | 76.1 | 33.1 | 109.1 | 51 | 181.5 ± 80.1 | 103.4 ± 42.6 | 285 ± 122.1 |
| LS | 8 | 40.7 ± 40.8 | 22.7 ± 21.1 | 55.8 ± 54.8 | 0 | 0 | 0 | 0 | 6 | 96.2 ± 77.6 | 57.4 ± 41 | 153.5 ± 118.4 |
| PH | 72 | 90 ± 50 | 28.4 ± 17.7 | 116.4 ± 65.7 | 18 | 51.7 ± 37.3 | 17.1 ± 17.3 | 65.9 ± 46.5 | 0 | 0 | 0 | 0 |
| PT | 107 | 38.1 ± 32.1 | 10 ± 8.5 | 47.6 ± 40.5 | 216 | 22.3 ± 15 | 6.2 ± 4.2 | 28.5 ± 19.1 | 1 | 15.3 | 5.7 | 21 |
| PS | 71 | 28 ± 23.5 | 7.9 ± 6.9 | 33.6 ± 28.3 | 0 | 0 | 0 | 0 | 0 | 0 | 0 | 0 |
| PAM | 39 | 38.7 ± 24.9 | 10.1 ± 6.5 | 45.4 ± 30.3 | 0 | 0 | 0 | 0 | 0 | 0 | 0 | 0 |
| RDA | 95 | 97.2 ± 64.4 | 27.8 ± 18.7 | 123.3 ± 82.4 | 24 | 51.1 ± 28.5 | 15.4 ± 9.2 | 66.5 ± 36.8 | 0 | 0 | 0 | 0 |
| RDM | 45 | 56.8 ± 59.8 | 16.3 ± 19.3 | 70.5 ± 77.4 | 14 | 40 ± 34.3 | 12 ± 8.7 | 52 ± 42.8 | 13 | 35.2 ± 38 | 10.8 ± 12 | 46 ± 50 |
| RDB | 15 | 39.2 ± 32.3 | 10.3 ± 8.2 | 45.7 ± 38.7 | 9 | 64 ± 50.7 | 21.1 ± 20.2 | 85.1 ± 69.1 | 1 | 16.4 | 4.4 | 20.8 |
| EUC | 11 | 69.4 ± 27 | 60.3 ± 67.1 | 122.3 ± 80.6 | 26 | 42.4 ± 27.3 | 42.6 ± 31.1 | 83.1 ± 46.2 | 0 | 0 | 0 | 0 |
| PPR | 22 | 93.7 ± 69 | 30.1 ± 21.3 | 114.4 ± 86.8 | 7 | 54.9 ± 29.2 | 15.4 ± 6.7 | 70.2 ± 35.8 | 3 | 16 ± 2.7 | 6.9 ± 1.4 | 22.9 ± 4 |
| PCF | 10 | 45.5 ± 34.8 | 24.1 ± 17 | 59.1 ± 48.5 | 28 | 45.9 ± 32.6 | 20.4 ± 16.8 | 66.4 ± 43.8 | 5 | 12.5 ± 13.6 | 9.3 ± 4.9 | 21.8 ± 17.4 |
| C | 5 | 80.4 ± 65.3 | 53.9 ± 41.8 | 127 ± 93.8 | 10 | 36.5 ± 24.3 | 23.7 ± 14.7 | 60.1 ± 38.7 | 0 | 0 | 0 | 0 |

| Forest type | | La Palma |  |  |  | El Hierro |  |  |
| --- | --- | --- | --- | --- | --- | --- | --- | --- |
|  | N | ACD | BCD | TCD | N | ACD | BCD | TCD |
| S | 0 | 0 | 0 | 0 | 14 | 13.7 ± 11 | 5.3 ± 3.7 | 18.9 ± 14.3 |
| ACL | 0 | 0 | 0 | 0 | 0 | 0 | 0 | 0 |
| P | 0 | 0 | 0 | 0 | 0 | 0 | 0 | 0 |
| FB | 71 | 79.6 ± 60.8 | 37.1 ± 34.1 | 116.6 ± 94.3 | 36 | 106 ± 56.2 | 54.4 ± 35.6 | 160.1 ± 90.1 |
| LH | 0 | 0 | 0 | 0 | 0 | 0 | 0 | 0 |
| L | 37 | 139 ± 60.1 | 69.7 ± 31.0 | 208.7 ± 90.5 | 1 | 186.3 | 89.8 | 275.9 |
| LS | 1 | 53.2 | 26.9 | 80.1 | 6 | 86.4 ± 50.2 | 46.6 ± 31.2 | 132.6 ± 81.3 |
| PH | 51 | 110.3 ± 63.5 | 38.2 ± 26.6 | 148.5 ± 87.8 | 0 | 0 | 0 | 0 |
| PT | 107 | 89 ± 55.2 | 23.9 ± 15.2 | 113 ± 70.3 | 39 | 68.5 ± 42.4 | 18.3 ± 11.4 | 86.8 ± 53.8 |
| PS | 53 | 57.4 ± 39.7 | 15.7 ± 10.4 | 73 ± 50 | 0 | 0 | 0 | 0 |
| PAM | 27 | 81.5 ± 46 | 22.9 ± 12.4 | 104.4 ± 58.4 | 0 | 0 | 0 | 0 |
| RDA | 0 | 0 | 0 | 0 | 18 | 63.5 ± 37.9 | 16.8 ± 9.9 | 80.2 ± 47.8 |
| RDM | 0 | 0 | 0 | 0 | 1 | 28.8 | 7.8 | 36.6 |
| RDB | 0 | 0 | 0 | 0 | 0 | 0 | 0 | 0 |
| EUC | 0 | 0 | 0 | 0 | 0 | 0 | 0 | 0 |
| PPR | 0 | 0 | 0 | 0 | 5 | 79.4 ± 95.5 | 33.1 ± 54.1 | 110.3 ± 145.1 |
| PCF | 3 | 83.3 ± 72 | 29.1 ± 26.5 | 112.4 ± 98.4 | 6 | 17 ± 13 | 4.7 ± 3.5 | 21.5 ± 16.4 |
| C | 4 | 66.3 ± 25.3 | 48.4 ± 24.7 | 114.6 ± 50 | 0 | 0 | 0 | 0 |

Table S8. Parametrization details of the calibration models for each island. Parameters: mtry = number of predictor variables randomly selected as candidates at each split in a decision tree, ntree = number of trees in the ensemble, OOB: out-of-bag error, interaction.depth= tree complexity, specifies the maximum depth of each individual tree, n.minobsinnode = minimum number of observations that must be present in a terminal node (leaf).

| Parametrization | Tenerife BRT | Gran Canaria BRT | La Gomera RF | La Palma RF | El Hierro RF |
| --- | --- | --- | --- | --- | --- |
| mtry | - | - | 10 | 5 | 6 |
| ntree | - | - | 500 | 500 | 500 |
| OBB | - | - | 5.904 | 2.292 | 2.590 |
| ntree | 6000 | 6000 | - | - | - |
| interaction.depth | 10 | 10 | - | - | - |
| n.minobsinnode | 5 | 10 | - | - | - |

Table S9. Results of validation of predictive models estimating total carbon densities (TCD) on forests of the five Canary Islands not counting with LIDAR data. Performance metrics for Random Forest (RF) models based on NFI plots (number of plots = n) are shown including the coefficient of determination (R²), Root Mean Squared Error (RMSE), Normalized RMSE% by range of observed values and Mean Absolute Error (MAE). Models were validated using 10-fold cross-validation.

|  | Model without LiDAR variables | | | | |
| --- | --- | --- | --- | --- | --- |
|  | RF | | | | |
| Island | R2 | RMSE | RMSE% | MAE | n |
| Tenerife | 0.56 | 50.91 | 11.63 | 36.76 | 524 |
| Gran Canaria | 0.35 | 28.83 | 15.69 | 19.91 | 289 |
| La Gomera | 0.74 | 81.51 | 11.19 | 54.11 | 153 |
| La Palma | 0.35 | 68.58 | 16.34 | 52.52 | 289 |
| El Hierro | 0.55 | 57.03 | 15.54 | 41.01 | 116 |

Table S10. Relative importance (%) of predictors in Random Forest (RF) models estimating total carbon densities (TCD) (above and belowground biomass) across NFI forest sites for five Canary Islands. Abbreviations of predictors follow table 1. LiDAR derived predictors (tree volume, height, cover and density) are not included. The importance value in RF reflects the increase in Mean Squared Error (%) compared to the mean importance.

| TFE |  | GC |  | LG |  | EH |  |
| --- | --- | --- | --- | --- | --- | --- | --- |
| VARIABLE | %IncMSE | VARIABLE | %IncMSE | VARIABLE | %IncMSE | VARIABLE | %IncMSE |
| vegtype | 12.815 | TIPO | 1.903 | TIPO | 12.260 | NDVI_win | 6.504 |
| dem | 4.462 | hum | 4.673 | NDVI_win | 16.386 | exp | 8.666 |
| NDVI_spr | 6.572 | NDVI_win | 6.509 | ndvi | 2.785 | pwet | 3.797 |
| pmed | 3.822 | pmed | 0.790 | hum | 4.537 | ndvi2020 | 7.234 |
| pdry | 7.303 | pdry | 12.591 | pmed | 11.058 | NDVI_spr | 4.868 |
| tmed | 7.588 | tseas | 4.455 | pdry | 4.901 | moist | 12.547 |
| tseas | 5.757 | b3 | 1.404 | pwet | 10.046 | b2 | 14.701 |
| tann | 4.273 | b4 | 0.481 | tmed | 4.954 | b3 | 3.124 |
| hum | 6.135 | b5 | 2.685 | tseas | 5.335 | b4 | 4.422 |
| moist | 19.825 | b11 | 5.647 | tann | 5.806 | b5 | -0.646 |
| NDVI_win | 10.984 | b12 | 4.442 | NDVI_spr | 1.839 | b6 | 3.582 |
| NDWI | 9.795 | moist | 5.608 | b2 | 5.951 | b7 | 5.339 |
| b2 | 9.243 | ndwi | 0.604 | b3 | 5.461 | b8 | 2.149 |
| b3 | 11.175 | NDVI_spr | 2.673 | b4 | 7.784 | b9 | 3.110 |
| b4 | 10.457 | trend | 17.634 | b5 | 4.535 | b11 | 4.665 |
| b5 | 8.805 |  |  | b6 | 2.317 | b12 | 12.517 |
| b6 | 6.406 |  |  | b7 | 5.657 | trend | 4.344 |
| b7 | 5.984 |  |  | b8 | 5.306 |  |  |
| b8 | 6.196 |  |  | b9 | 5.279 |  |  |
| b9 | 5.538 |  |  | b11 | 2.243 |  |  |
| b11 | 10.041 |  |  | b12 | 11.896 |  |  |
| b12 | 18.846 |  |  | moist | 13.105 |  |  |
| trend | 8.137 |  |  | trend | 2.817 |  |  |

Source of data from National Forest Inventory of Spain:

https://www.miteco.gob.es/es/biodiversidad/temas/inventarios-nacionales/inventario-forestal-nacional/cuarto_inventario.html

*References*

BEI group. Research group Island Biogeography and Ecology, University of La Laguna, Tenerife, Spain.

Burrows, L., E. Cieraad, and N. Head. Scotch broom facilitates indigenous tree and shrub germination and establishment in dryland New Zealand. *New Zealand Journal of Ecology* 2015; 39: 61-70.

Issa S, Dahy B, Saleous N, Ksiksi T, et al. Carbon stock assessment of date palm using remote sensing coupled with field-based measurements in Abu Dhabi (United Arab Emirates). *Int J Remote Sens* 2019; 40: 7561-7580. [https://doi.org/10.1080/01431161.2019.1602795](https://doi.org/10.1080/01431161.2019.1602795)

Menéndez-Miguélez M, Ruiz-Peinado R, del Río M, Calama R, et al. Improving tree biomass models through crown ratio patterns and incomplete data sources. *Eur J For Res*. 2021; 140:675-689.

MITECO. *Ecuaciones alométricas de las especies arbóreas de Canarias. Canarias*. Ministerio para la Transición Ecológica y el Reto Demográfico (unpbl. Data 2021).

Montero G, López-Leiva C, Ruiz-Peinado R, López-Senespleda E, Onrubia R, Pasalodos M. Producción de biomasa y fijación de carbono por los matorrales españoles y por el horizonte orgánico superficial de los suelos forestales. Madrid: INIA; 2020.

Montero, G., R. Ruiz-Peinado, and M. Muñoz. *Producción de Biomasa y Fijación de CO_2_ por los Bosques Españoles*. Ministerio de Educación y Ciencia, Madrid, España. 2005.

Ruiz-Peinado R, Moreno G, Juarez E, Montero G, Roig S, et al. The contribution of two common shrub species to aboveground and belowground carbon stock in Iberian dehesas. *J Arid Environ*. 2013; 91:22-30.

Vega, AP. La biomasa subterránea en los matorrales canarios: revisión bibliográfica y estimaciones preliminares. Trabajo fin de grado. Universidad de la Laguna. 2023.
