# Supplementary material for: Modelling forest carbon stocks on the Canary Islands
Source: Carbon Balance Manag. 2026 Jul 27;21:104. doi: 10.1186/s13021-026-00488-4 (PMC13452119; doi:10.1186/s13021-026-00488-4)
Supplement: Supplementary file 2 [file 13021_2026_488_MOESM2_ESM.docx]

**Figures Supplementary**

Fig. S1. Distribution of forest types on the Canary Islands. S: Juniper woodlands, P: Palm forest, FB: *Erica-Morella* forest, L: Typical laurel forest, LS: Dry laurel forest, LH: Humid laurel forest, PS: Dry Canarian pine forest, PT: Typical Canarian pine forest, PH: Humid Canarian pine forest, PAM: High-elevation Canarian pine forest, RDB: Low-density plantations of Canarian pine, RDM: Medium-density plantations of Canarian pine, RDA: High-density plantations of Canarian pine, PCF: Plantations of exotic conifers and broad-leaved species, PPR: Plantations of *Pinus radiata,* EUC: Plantations of *Eucalyptus* species, C: Plantations of chestnut (*Castanea sativa*), ACL: Wild Canarian olive tree woodlands. Comunidades: forest type.

Gran Canaria

Tenerife

La Palma

La Gomera

El Hierro

Fig. S2. Distribution of NFI plots on the Canary Islands. S: Juniper woodlands, P: Palm forest, FB: *Erica-Morella* forest, L: Typical laurel forest, LS: Dry laurel forest, LH: Humid laurel forest, PS: Dry Canarian pine forest, PT: Typical Canarian pine forest, PH: Humid Canarian pine forest, PAM: High-elevation Canarian pine forest, RDB: Low-density plantations of Canarian pine, RDM: Medium-density plantations of Canarian pine, RDA: High-density plantations of Canarian pine, PCF: Plantations of exotic conifers and broad-leaved species, PPR: Plantations of *Pinus radiata,* EUC: Plantations of *Eucalyptus* species, C: Plantations of chestnut (*Castanea sativa*), ACL: Wild Canarian olive tree woodlands. Parcelas de biomasa: IFN plot.

Gran Canaria

La Palma

El Hierro

Tenerife

La Gomera

Fig. S3. Correlation matrix of 29 continuous predictor variables. Abbreviations of predictors see Table 1.

Fig. S4. Total carbon stock and mean total carbon density (above- and belowground) across the 18 forest types on the Canarian archipelago (all islands included).

Fig. S5. Spatial distribution of forest aboveground carbon density (ACD, Mg C ha^−1^) for each Canarian Island at 50-m mapping resolution. Best models used to calculate total carbon stocks included Boosted Regression Tree (BRT) and Random Forest (RF). Observe that the scales used in the different islands are variable.

Fig. S6. Uncertainty maps of total forest carbon density (TCD, Mg C ha^−1^) for each Canarian Island at 50-m mapping resolution. Best models used to calculate total carbon stocks included Boosted Regression Tree (BRT) or Random Forest (RF). Observe that the scales used in the different islands are variable.

Fig. S7. Uncertainty maps of total aboveground carbon density (TCD, Mg C ha^−1^) for each Canarian Island at 50-m mapping resolution. Best models used to calculate total carbon stocks included Boosted Regression Tree (BRT) or Random Forest (RF). Observe that the scales used in the different islands are variable.
